# Ligand vacancy channels in pillared inorganic-organic hybrids for electrocatalytic organic oxidation with enzyme-like activities

**DOI:** 10.1038/s41467-023-36830-4

**Published:** 2023-03-02

**Authors:** Zhe Chen, Jili Li, Lingshen Meng, Jianan Li, Yaming Hao, Tao Jiang, Xuejing Yang, Yefei Li, Zhi-Pan Liu, Ming Gong

**Affiliations:** 1 Department of Chemistry and Shanghai Key Laboratory of Molecular Catalysis and Innovative Materials, Fudan University, Shanghai, 200438 China; 2 National Engineering Laboratory for Industrial Wastewater Treatment, East China University of Science and Technology, Shanghai, 200237 China

**Keywords:** Electrocatalysis, Electrocatalysis, Energy, Electrocatalysis

## Abstract

Simultaneously achieving abundant and well-defined active sites with high selectivity has been one of the ultimate goals for heterogeneous catalysis. Herein, we construct a class of Ni hydroxychloride-based inorganic-organic hybrid electrocatalysts with the inorganic Ni hydroxychloride chains pillared by the bidentate N-N ligands. The precise evacuation of N-N ligands under ultrahigh-vacuum forms ligand vacancies while partially retaining some ligands as structural pillars. The high density of ligand vacancies forms the active vacancy channel with abundant and highly-accessible undercoordinated Ni sites, exhibiting 5-25 fold and 20-400 fold activity enhancement compared to the hybrid pre-catalyst and standard β-Ni(OH)_2_ for the electrochemical oxidation of 25 different organic substrates, respectively. The tunable N-N ligand can also tailor the sizes of the vacancy channels to significantly impact the substrate configuration leading to unprecedented substrate-dependent reactivities on hydroxide/oxide catalysts. This approach bridges heterogenous and homogeneous catalysis for creating efficient and functional catalysis with enzyme-like properties.

## Abstract (web summary)

Undercoordinated metal sites with well-defined structures and stability are desired properties for catalysts. Here, the authors construct a class of inorganic-organic hybrid electrocatalysts with enzyme-like properties, which exhibit enhanced electrochemical oxidation of 25 different organics.

## Introduction

Undercoordinated metal sites are crucial to heterogeneous catalysis^1–3^. The termination of heterogeneous surfaces with surface sites that are undercoordinated or weakly coordinated to atmospheric species creates the sites for substrate adsorption and activation. Artificial incorporation of defective sites with undercoordination characteristics via chemical reduction^4–7^, plasma treatment^8–11^ and etching^12–15^ has been a great success in enhancing the catalytic activity. However, the structural complexity of these defective surfaces has led to significant difficulties in pinpointing and maximizing the real active sites. Comparatively, catalysts with isolated metal sites embedded in scaffolds, such as metal organic frameworks (MOFs)^16–18^, zeolites^19–21^ and carbon matrixes^22–25^, as well as metalloenzymes^26–28^ could narrow down the structural distribution of the metal sites, allowing for the identification of the chemical identities for the undercoordinated metal sites. These isolated sites effectively improve the selectivity via eliminating the side pathways involving multiple metal centers and also significantly enhance the catalytic turnovers per metal site, but meanwhile demand burdensome scaffolds to confine the metal sites, and thus lacking sufficient metal centers for high gravimetric catalytic activities^29,30^. Therefore, simultaneously achieving abundant undercoordinated sites with well-defined structures has been one of the ultimate goals for efficient heterogeneous catalysis.

Pillared inorganic-organic hybrid materials, often consisting of inorganic layers or chains and axially-coordinated organic ligands, have attracted emerging attention owing to the structural tunability of pillared organic ligands that give rise to their unique properties^31–34^. Specifically, hybrid compounds with a formula of MX_2_(N-N) (M = Fe, Co, Ni; X = Cl, Br; N-N = bidentate organic linkers, such as pyrazine, 4,4’-bipyridine, *trans*−4,4’-azopyridine and etc.) are a class of inorganic-organic hybrids with excellent magnetic properties^31,35–38^. In the MX_2_(N-N) structures, the M centers are octahedrally coordinated by four X ligands and two N atoms from the bidentate ligand in a trans disposition. The X ligands bridge the metal centers along one direction to form an inorganic chain, while the N-N bidentate ligand pillars the inorganic chain forming an organic channel. The tunable N-N ligands could effectively modify the electronic properties of the metal centers to tailor their magnetic properties. The catalytic use of the MX_2_(N-N) materials has been previously restricted due to the limited stability in polar solvents and their saturated coordination. However, the abundant metal centers as well as the tunable metal-ligand coordination therein could potentially enable molecular engineering for efficient catalysis with enriched and undercoordinated metal sites.

Herein, we discovered a structural analog to the NiCl_2_(N-N) material via partially substituting the bridging chlorides with hydroxides. The replacement by hydroxides drastically enhances its structural integrity for preventing its dissolution in aqueous conditions. Further partial removal of the N-N ligands created ligand vacancy sites, and a high level of adjacent vacancy sites form the vacancy channels with enriched undercoordinated metal sites, allowing for the substrate diffusion and catalytic turnover. Taking the Ni hydroxychloride (NiHC)-pz (pz=pyrazine) material as an example, the ultrahigh vacuum annealing process evacuated the pyrazine ligands without structural collapse and ligand fragmentation, and the catalyst with vacancy channels exhibited significantly higher gravimetric activities compared to the parent NiHC-pz and the standard β-Ni(OH)_2_ material for the electrocatalytic oxidation of various organic molecules, including urea, alcohols, aldehydes and amines. This approach could be generalized to other Ni-based inorganic-organic hybrids, and the different sizes of ligand vacancy channels for size-dependent activities of selective substrate oxidation, mimicking the enzymatic behaviors but with greatly accelerated rates.

## Results

### Synthesis and characterization of NiHC-pz catalysts

**Fig. 1:**
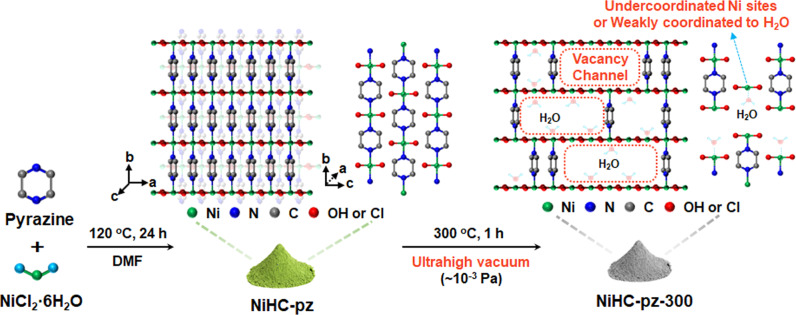
Schematic illustration of the catalyst preparation and structure. Schematic illustration of the synthesis and structure of NiHC-pz and NiHC-pz-300.

**Fig. 2:**
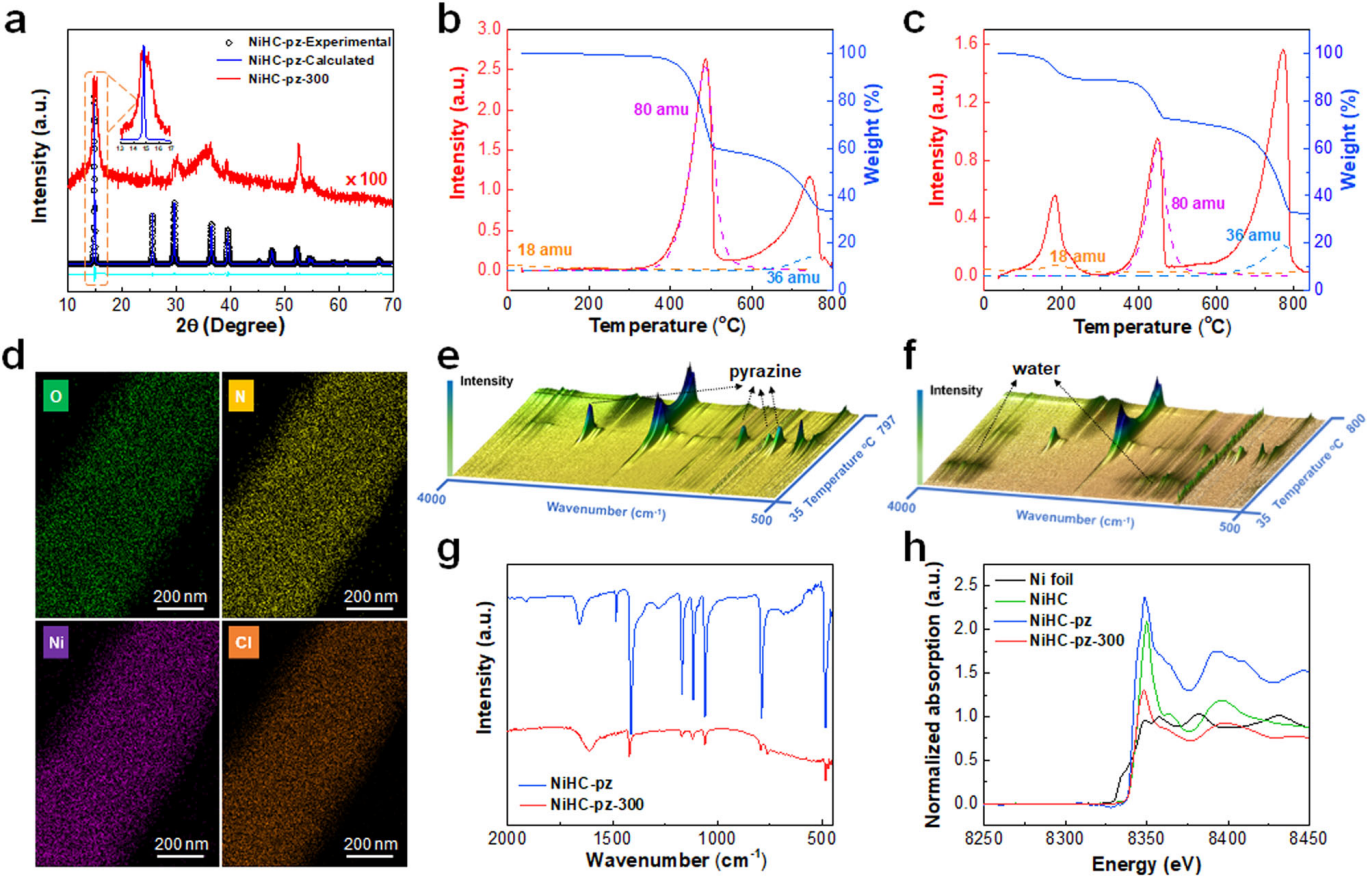
Characterization of NiHC-pz catalysts. **a** The PXRD pattern of the NiHC-pz and NiHC-pz-300, and the inset shows the magnified view of the (110) peak. **b** The FTIR spectra of the NiHC-pz and NiHC-pz-300. **c** The XANES spectra of the Ni foil, NiHC, NiHC-pz and NiHC-pz-300. **d** TG spectrum and MS counting of the NiHC-pz. **e** TG spectrum and MS counting of the NiHC-pz-300. **f** FTIR spectra of the NiHC-pz. **g** FTIR spectra of the NiHC-pz-300 for the TG-FTIR-GCMS analysis. **h** EDX mapping of the NiHC-pz-300.

The NiHC-pz pre-catalyst was synthesized via a solvothermal method at 120 ^o^C in a N,N-dimethylformamide (DMF)/H_2_O mixed solution (Fig. 1). The presence of H_2_O partially hydrolyzed the NiCl_2_ to form bridging hydroxide analogous to Ni(OH)_2_. Since the hydroxide ligands share structural similarity to the chloride ligands, the X-ray diffraction (XRD) pattern of the NiHC-pz material highly resembled that of the NiCl_2_-pz material with sharp diffraction peaks at 2*θ* angles of 14.68°(major), 25.43°, 29.46°, 36.32° and 39.34° (Fig. 2a)^39^. The Rietveld refinement of the XRD pattern corresponded to an orthorhombic cell (space group: Cmmm) with Ni centers octahedrally coordinated to four hydroxide or chloride ligands and two axial nitrogen atoms from the pyrazine ligands. The pyrazine ligands pillar the Ni hydroxide/chloride chains along the *b* direction, while each chain slides in the *b* direction and stacks in an ABAB manner along the *c* direction (Fig. 1). The hydroxide ligands could not be precisely located via refinement, but their presence was clearly evidenced. The material synthesized without pyrazine ligands exhibited a highly consistent XRD pattern with the reported Ni hydroxychloride material (Supplementary Fig. 1)^40^. Meanwhile, the O content in NiHC-pz according to the X-ray photoelectron spectroscopy (XPS) could reach an almost identical value compared to Cl (Supplementary Fig. 2), and the O, N and Cl elements shared similar spatial distribution according to the energy dispersive X-ray spectroscopy (EDX) mapping (Supplementary Fig. 3).

The hydroxide incorporation greatly enhances the structural integrity, preventing significant dissolution in aqueous solution. This could allow the evacuation of pyrazine for creating undercoordinated Ni sites. We utilized the thermogravimetric analyzer coupled with Fourier transform infrared spectrometry and gas chromatography-mass spectrometry (TG-FTIR-GCMS) to evaluate the dynamic structural evolution during the thermal decomposition. The NiHC-pz was thermally stable up to 350 ^o^C and then subject to two decomposition stages at 350–500 ^o^C and > 500 ^o^C respectively (Fig. 2b). The first decomposition stage only involves the loss of pyrazine ligand, as indicated by the 80 amu peak in the MS spectrum and the emerging FTIR peaks at 783, 1028, 1135 and 1411 cm^−1^ (Fig. 2b, e). The loss of pyrazine ligand corresponds to ~40.4 wt% loss of the NiHC-pz, pointing to the stoichiometry of Ni(OH)Cl-pz. After the pyrazine loss, the material further decomposes with the evolution of HCl as indicated by the 36 amu peak. The presence of H in the evolved HCl gas supports the partial incorporation of hydroxide in NiHC-pz (Fig. 2b). During the decomposition process, negligible loss of H_2_O (18 amu) was observed, excluding the possibility of Ni(OH)_2_ phase and a large extent of adsorbed or hydration H_2_O.

Despite the creation of pyrazine vacancies, the NiHC-pz annealed at 400 ^o^C under N_2_ produced surface carbonaceous species that complicates and blocks the active site (Supplementary Fig. 4). Instead, we utilized an ultrahigh vacuum system at 10^−3^ Pa to thermodynamically drive the ligand evacuation to lower temperatures. The annealing at 300 ^o^C under ultrahigh vacuum could simultaneously achieve the steady loss of pyrazine ligands and prevent the downstream side-reactions of pyrazine decomposition or carbonization. According to the TG analysis, the 350–500 ^o^C decomposition stage exhibited significantly lower weight loss, corresponding to 30% of the pyrazine remaining (Fig. 2c). These pyrazine ligands could serve as pillars to maintain the structural integrity from devastating collapse, while 70% of the pyrazines form vacancy sites and a high possibility of adjacent vacancy sites creates the vacancy channel (Fig. 1), allowing alien substrate molecules to diffuse into the channels for reacting with the undercoordinated Ni sites. Interestingly, an additional decomposition stage that corresponds to ~11.4 wt% loss appeared before 300 ^o^C, and the MS and FTIR spectra confirmed its contribution from H_2_O (Fig. 2c, f). The introduction of H_2_O was presumably stemmed from the hygroscopic nature of the undercoordinated Ni sites, which in turn endorsed the vacancy sites inside the channels. Further TEM analysis revealed that compared to the rod-like structures with smooth edges of the NiHC-pz pre-catalyst, the NiHC-pz-300 catalyst mostly maintained the macroscopic rod-like structures with uniform Ni, O, N and Cl distribution but with roughened edges (Fig. 2d and Supplementary Fig. 5), possibly due to the pyrazine loss as resolved by FTIR with lower intensities (Fig. 2g).

The XRD patten of the NiHC-pz-300 mostly maintained the NiHC-pz structure but with a significantly decreased signal due to the loss of lattice symmetry from the pyrazine evacuation (Fig. 2a). Comparatively, the NiCl_2_(pz) without hydroxide substitution, although with similar structure and elemental distribution (Supplementary Fig. 6), was completely collapsed upon annealing to NiCl_2_ that was hygroscopic and could readily dissolve in water, further supporting the enhanced integrity via hydroxide incorporation (Supplementary Figs. 7, 8). Importantly, the NiHC-pz-300 demonstrated additional peaks that shifted toward higher angles for the (110) and (111) peaks but not for the (001) peak (Fig. 2a and inset). The (001) peak was indicative of the lattice along the NiHC chain, implying that upon annealing the NiHC chain remained intact while slightly shrinking the lattice along the other directions due to the pyrazine evacuation to stabilize the structure. Despite the slightly shrunk lattice, the structure maintained the vacancy channels without the structural devastation as in the case of the NiCl_2_(pz) material. The X-ray absorption near-edge structure (XANES) spectra revealed the mostly maintained Ni(II) characteristics due to the charge-neutral pyrazine ligands (Fig. 2h)^41,42^. The XPS analysis also confirmed the major Ni^+2^ oxidation state at 855.5 eV, but upon pyrazine evacuation, the Ni^+2^ peak was slightly broadened compared to the pristine material, which indicated an increased heterogeneity with local coordination environment changes (Supplementary Fig. 2). These changes also affected the local coordination environment of chloride and hydroxide by showing the broadened Cl 2p peak and shifted M-OH peak toward larger O 1 s binding energy in XPS (Supplementary Fig. 2). All of these characterization results point to the creation of pyrazine vacancy channels and undercoordinated Ni sites without structural collapse in the NiHC-pz hybrid, which sets up the basis for its catalytic application.

### Electrochemical oxidation of organics with ultrafast turnovers

Ni-based catalysts, especially Ni oxyhydroxide, can electrochemically oxidize various organics (such as alcohols, aldehydes and amines) with high selectivity to the corresponding carboxylic acids and nitriles^43–45^. Pairing the anodic organic oxidation with cathodic hydrogen evolution reaction could simultaneously achieve pure hydrogen production with lower energy demands and the production of value-added organic molecules, which is often considered as a promising next-generation hydrogen-production technology^46^. The oxidation activity is not only pertinent to the intrinsic Ni coordination environment but also often governed by the accessibility of the Ni sites, which could be highly beneficial for the vacancy channel in NiHC-pz-300. Therefore, the electrochemical oxidation activities of 25 organic substrates were evaluated by polarization curves at slow scan rates in 1 M KOH with 0.1 M substrate (Supplementary Fig. 17). These organic substrates span from alcohols, aldehydes and amines to compounds with multiple reactive centers (such as diols, polyols and urea). The NiHC-pz-300 catalyst at 1 mg_cat_/cm^2^ mostly showed rapidly-growing currents after Ni oxidation and quickly entered the diffusion-controlled stage, demonstrating the exceptional electrocatalytic activities that quickly deplete the substrates near the active sites (Supplementary Figs. 9–17).

**Fig. 3:**
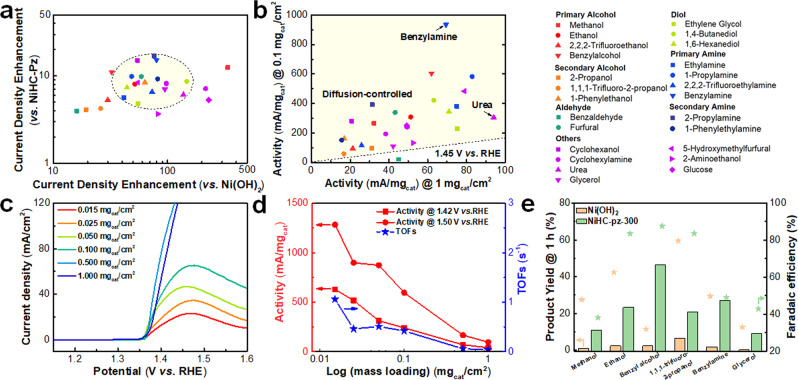
Electrochemical oxidation of organics with ultrafast turnovers. **a** The current density enhancement of NiHC-pz-300 compared to NiHC-pz and β-Ni(OH)_2_ from the electrolytic current density at 1.45 V *vs*. RHE for 25 different substrates (0.1 M substrate in 1 M KOH). **b** The gravimetric current densities of NiHC-pz-300 at the loadings of 0.1 mg_cat_/cm^2^ and 1 mg_cat_/cm^2^ for 25 different substrates showing diffusion-controlled kinetics. **c** The polarization curves of NiHC-pz-300 under different loadings in 1 M KOH + 0.1 M urea. **d** The gravimetric current densities of NiHC-pz-300 at different loadings at 1.42 V *vs*. RHE and 1.50 V *vs*. RHE in 1 M KOH + 0.1 M urea, and the calculated TOF (1.50 V) values under different loadings. **e** The product yields and corresponding faradaic efficiencies of Ni(OH)_2_ (1 mg_cat_/cm^2^) and NiHC-pz-300 (1 mg_cat_/cm^2^) in 1 M KOH and 0.1 M substrates electrolyzed at 1.45 V *vs*. RHE for 1 h (except 1.50 V *vs*. RHE for 1,1,1-trifluoro-2-propanol).

To more precisely evaluate its intrinsic activity, we lowered the catalyst mass loading to 0.1 mg_cat_/cm^2^ and compared with the standard catalysts of NiHC-pz and β-Ni(OH)_2_. We also measured the chronoamperometric curves without any iR compensation to minimize the contribution from non-Faradaic current, We plotted the activity enhancement factor of the electrolytic current density as well as the current density (@1.45 V *vs*. reversible hydrogen electrode (RHE), mA/mg_cat_) *vs*. NiHC-pz and β-Ni(OH)_2_ respectively, and discovered that for most organic substrates we could achieve 5–17 fold activity increase *vs*. NiHC-pz and 40–150 fold increase *vs*. Ni(OH)_2_ (Fig. 3a, b and Supplementary Figs. 18–25) for the electrolytic current, while achieving 5–25 fold *vs*. NiHC-pz and 20–400 fold *vs*. Ni(OH)_2_ from the values from the polarization curves (Supplementary Fig. 17a, b). By analyzing the activity trend, we found that smaller molecules typically exhibited higher activities; for instance, the activity on NiHC-pz-300 showed a much decrease from ethanol to trifluoroethanol, whereas this decrease was much less obvious on NiHC-pz (Supplementary Fig. 9). This was mostly attributed to more exposure of undercoordinated Ni sites inside the vacancy channels that created diffusion obstacles for sterically-hindered substrates.

The highest gravimetric activity was achieved on urea with 518.5 mA/mg_cat_ @ 1.42 V *vs*. RHE (Supplementary Fig. 17b). Urea electrolysis is a promising technique for the simultaneous hydrogen production and urea abatement, and an efficient urea oxidation catalyst is often a prerequisite for on-site urine treatment^47–49^. By using urea as a typical substrate, we further analyzed the electrochemical oxidation kinetics on our NiHC-pz-300 catalyst. The superior activity of NiHC-pz-300 led to no current decrease when catalyst loading was halved from 1.00 mg_cat_/cm^2^ to 0.50 mg_cat_/cm^2^, supporting the diffusion-controlled kinetics (Fig. 3c). Even if the loading was lowered to 0.05 mg_cat_/cm^2^, the LSV curve greatly overlapped at the current onset where the kinetic-controlled region often resides. The Tafel plots displayed two regions with one rapid Ni oxidation region (Tafel slopes of 10.5–12.4 mV/decade) intimately followed by a diffusion-controlled region (Tafel slopes of > 120 mV/decade) at all loadings (Supplementary Fig. 26). The absence of a kinetic-controlled region indicated the high turnover activities of the undercoordinated Ni sites, and the reaction rates were mostly governed by the rates of substrate diffusion into the active vacancy channels. This phenomenon was typically existent in the metalloenzymes, with highly active metal centers buried inside protein scaffolds. We further utilized the steady-state Faradaic current density of urea oxidation to plot the gravimetric activity over catalyst loading and demonstrated a steady activity increase at lower loadings, with maximal activities of 630.67 mA/mg_cat_ @1.42 V *vs*. RHE and 1283.33 mA/mg_cat_ @1.50 V *vs*. RHE (Fig. 3d and Supplementary Fig. 27). We also measured the electrochemically accessible Ni sites via the Ni redox peak (Supplementary Fig. 28), and clearly resolved larger redox peaks than NiHC-pz and Ni(OH)_2_. As for the electrodes under different loadings, we could observe almost linearly-scaled electrochemical surface areas on loadings (Supplementary Fig. 29), while the Faradaic current density exhibited a non-linear response, suggesting higher turnover frequencies (TOFs) at low loadings. We calculated the TOFs at 1.50 V *vs*. RHE (Fig. 3d) and derived the highest value of 1.06 s^−1^ (based on 6 electron transfers of urea into dinitrogen), outperforming traditional heterogeneous catalytic oxidation reactions and approaching the enzymatic activities of dehydrogenases for selective oxidation^50–54^. We also extended the study to other representative substrates and carried out the liquid product analysis by NMR (Fig. 3e and Supplementary Tables 1–3). Compared to the standard Ni(OH)_2_ catalysts, the NiHC-pz-300 exhibited consistently higher liquid product yields of the corresponding ketones or carboxylates at 1.45–1.50 V *vs*. RHE for 1 h electrolysis. When the catalyst loading was further lowered to 0.1 mg/cm^2^, the electrolytic current and product yield were only slightly lower, consistent with the aforementioned results (Supplementary Tables 1–3). The detailed analysis of the Faradaic efficiencies (FEs) for the liquid products revealed that FEs were similar for most substrates (Fig. 3e and Supplementary Tables 1–3), implying that the active sites within the catalyst structures were rather similar and the activity enhancement was greatly stemmed from the drastically higher accessibility of the active sites within the ligand cavity channels. Further catalytic stability at 20 mA/cm^2^ showed relatively stable potential profiles with only a decay of 66 mV over 60 h (Supplementary Fig. 30). This activity decay was also present and even larger on NiHC-pz and β-Ni(OH)_2_ with drastically decayed activity over repetitive runs or gradually-decayed activity over the first few hours while this decay was intrinsic to the urea oxidation but not catalyst quality due to the stable oxygen evolution reaction (Supplementary Fig. 31), implying that the decay of NiHC-pz-300 was not stemmed from the structural collapse but instead possibly from the structural reconstruction under applied potentials.

### Highly accessible Ni sites via vacancy channels and dynamic ligand exchange

**Fig. 4:**
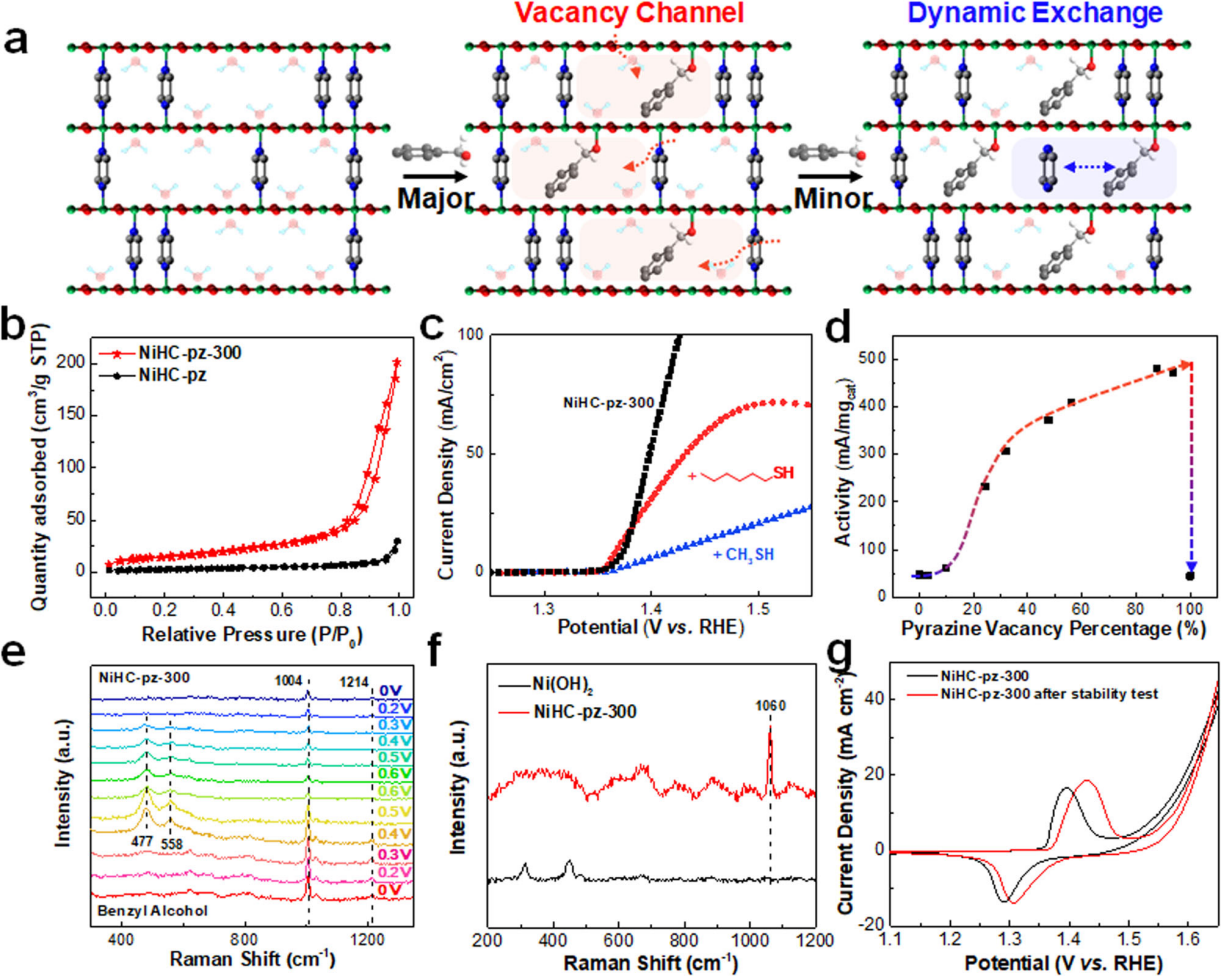
Highly accessible Ni sites via vacancy channels and dynamic ligand exchange. **a** Schematic illustration of the reaction pathways in NiHC-pz-300. **b** The N_2_ sorption isotherms of NiHC-pz and NiHC-pz-300. **c** The polarization curves of the NiHC-pz-300, methyl mercaptan poisoned NiHC-pz-300 and *n*-hexyl mercaptan poisoned NiHC-pz-300 in 1 M KOH + 0.1 M urea. **d** The structural-activity correlation of the gravimetric current density at 1.44 V *vs*. RHE vs. pyrazine vacancy percentages, showing positive but non-linear correlations. **e** The in situ Raman spectra of the NiHC-pz-300 under different applied potentials in 1 M KOH + 0.1 M benzyl alcohol. **f** The Raman spectra of the β-Ni(OH)_2_ and NiHC-pz-300 material removed from the electrolyte and washed with water after immersing with benzyl alcohol. **g** The CV curves of NiHC-pz-300 in 1 M KOH before and after the stability test.

The superior electrochemical activity of NiHC-pz-300 drove us to further glean the correlation between the pyrazine vacancies at the undercoordinated Ni sites and the electrocatalytic activities. Upon pyrazine evacuation, the NiHC-pz-300 demonstrated the typical IV N_2_ isotherm curves with a BET surface area of 52.32 m^2^ g^−1^, which is almost 5.5 times than NiHC-pz pre-catalyst (9.42 m^2^ g^−1^) (Fig. 4b). The pore size distribution curves revealed the presence of mesopores (2.4–49.6 nm) in the NiHC-pz-300 (Supplementary Fig. 32), supporting the formation of open spaces for facilitating the substrate diffusion and subsequent electrocatalysis. Since both outer surface sites and inner pore sites could contribute to the electrocatalytic activity, we then utilized poison molecules to differentiate the surface sites and the vacancy sites in the active channel (Fig. 4c). Methyl mercaptan was utilized to poison both surface sites and vacancy sites, while hexyl mercaptan was utilized to poison only surface sites due to its larger size. Successful incorporation of the thiols was evidenced by the evolution of C-H vibration and stretching bands at 2924.6, 2915.9, and 2846.4 cm^−1^ in FTIR, while the pyrazine vibrations were also enhanced with the thiols (Supplementary Fig. 33). Consistent with our expectations, the methyl mercaptan drastically decreased the current, whereas only a slight activity decay was resolved for hexyl mercaptan due to surface poisoning, advocating the major reaction environment at the vacancy channels (Fig. 4c). We further systematically tuned the number of pyrazine vacancy sites via controlling annealing conditions (e.g., lowered the reaction temperature to 275 ^o^C and controlled the reaction durations from 15 min to 1 h) to investigate the intrinsic structural-activity correlation pertinent to the vacancy numbers. The remained pyrazine ligands were estimated by dissolving the materials in *aqua regia* and quantifying the pyrazines by nuclear magnetic resonance (NMR) (Supplementary Fig. 34). According to the derived structural-activity correlation, pyrazine vacancies were first crucial by showing inferior activities at no pyrazine vacancies and full removal of pyrazines that collapses the structure (Fig. 4d). At relatively low pyrazine vacancy densities of < 10%, we did not observe instantaneous activity increases presumably due to the disconnected vacancy sites that obstruct the substrate diffusion. Increasing the ratio beyond 10% induced a rapid increase of the activity, while this increase gradually levelled off upon increasing vacancy numbers. This counter-intuitive non-linear scaling relationship might suggest that there exists another reaction pathway associated with the pyrazine. According to the monodentate Ni-pyrazine coordination, we envision that some of the remaining structural pyrazines might dynamically exchange with the substrates during catalysis to access the non-vacancy sites once some vacancies were first created as the entry sites.

To verify our hypothesis, we conducted in situ Raman spectroscopy using NiHC-pz-300 (Fig. 4e). Benzyl alcohol was utilized as the probe molecule due to its strong aromatic bands at ~1004 cm^−1^. The pristine NiHC-pz-300 exhibited a weak signal at 1204 cm^−1^, corresponding to the C-H bending mode ν_3_ in pyrazine^55^. Upon applying anodic potentials beyond the Ni oxidation, we observed the evolution of e_g_ bending mode and A_1g_ stretching mode of Ni^III^-O at 477 and 558 cm^−1^ resembling the bands in NiOOH^56,57^, accompanied by the vanishing pyrazine bands (Fig. 4e). Reverting the potential polarity fully recovered Ni-O bands and partially recovered the pyrazine bands. It is worth to note that for every repetitive runs, the N^III^-O bands were completely removed upon reduction, which could be barely achieved on conventional Ni(OH)_2_ surfaces^58^ and corroborate the highly accessible Ni sites in NiHC-pz-300. The reversible pyrazine loss and recovery cycle advocates that the pyrazines can dynamically exchange with the substrates for accessing more Ni sites. It has been previously reported that the Ni(OH)_2_ showed a lattice expansion via intercalating alien species (e.g., K^+^ cation)^59^ upon oxidation, and we speculate that this lattice expansion might also exist in NiHC-pz-300 so that the Ni-pyrazine coordination could be weakened for the dynamic ligand exchange. This was also evidenced by the loss of initial pyrazine peaks and the evolution of some subtle peaks at similar positions in FTIR after electrolysis (Supplementary Fig. 35). Similarly, partial N 1 s XPS signal was also still observed after the catalytic reactions (Supplementary Fig. 36). In spite of the dynamic coordination, we did not observe significant structural collapses and rapid activity decays. We render that the substrates could serve as alternative pillars to maintain the active channels for catalysis. Consequently, we examined the tested material with complete electrolyte removal. The presence of aromatic bands from benzyl alcohols was clearly resolved, indicating the trapped benzyl alcohols in the interspaces of NiHC-pz-300 (Supplementary Fig. 37). In addition, the ex situ Raman study indicated the much facilitated trapping of the benzyl alcohols inside the NiHC-pz-300 compared to the standard Ni(OH)_2_ catalyst (Fig. 4f), further supporting the feasibility of using trapped substrate for the catalytic reaction and alternative structural pillar. We also compared the Ni redox in the CV curves of the catalyst electrode before and after the electrolysis, and showed an increasing density of electrochemically-accessible Ni sites from 3.93 µmol/cm^2^ to 5.85 µmol/cm^2^ (Fig. 4g), which further evidenced the dynamic coordination and the absence of structural collapse. The slight shift of the Ni redox toward more positive potentials implied the changes in the local coordination environment with the possible partial loss of pyrazine (Fig. 4e, f). Therefore, the vacancy channels in NiHC-pz-300 can not only create well-accessible undercoordinated Ni sites for catalysis but also provide entry points for substrates to dynamically exchange with the pillared pyrazines for fully maximizing the activity (Fig. 4a).

### Tunable channels for size-selective electrochemical oxidation

**Fig. 5:**
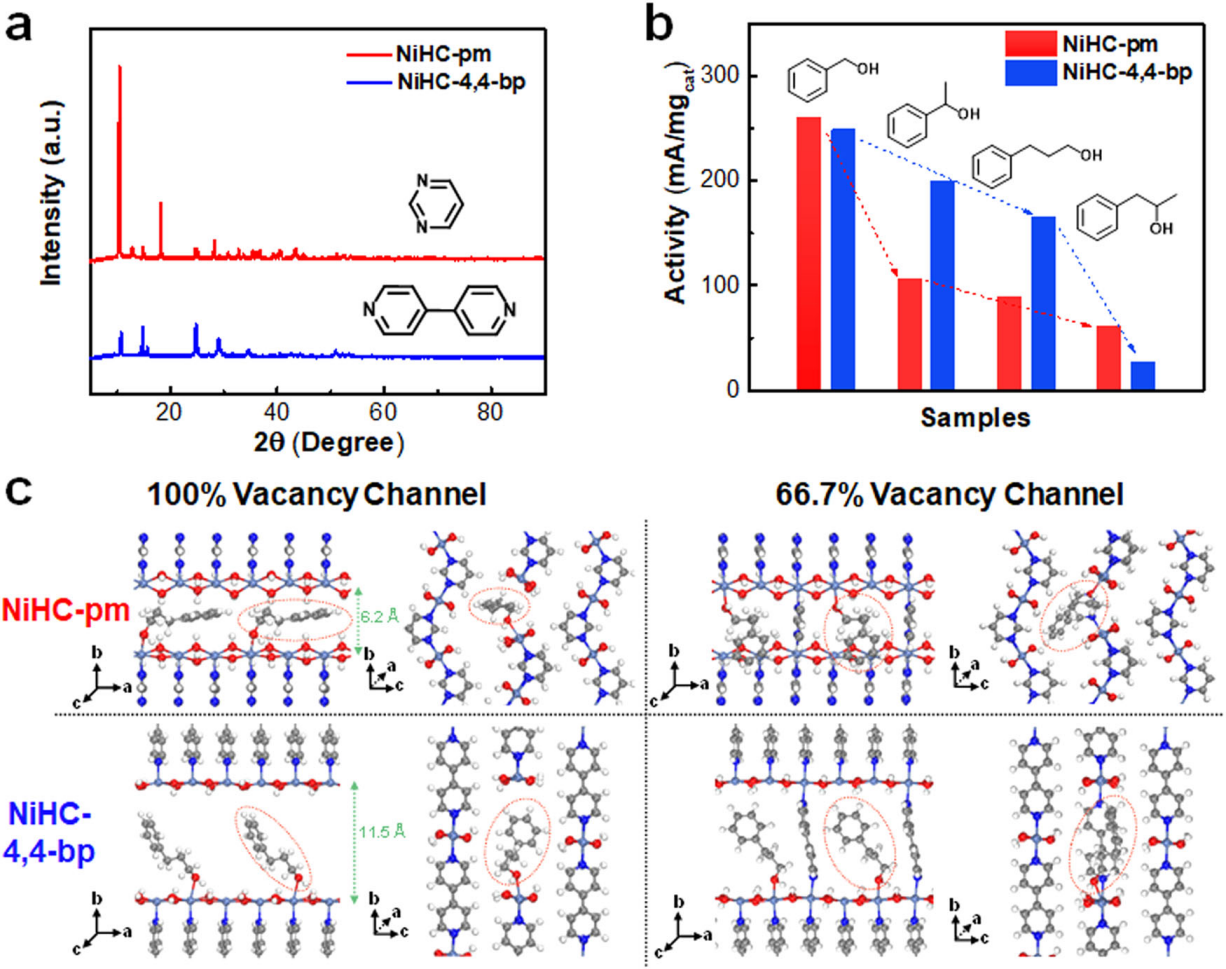
Tunable channels for size-selective electrochemical oxidation. **a** The PXRD patterns of the NiHC-pm and NiHC-4,4-bp. **b** The gravimetric current densities of NiHC-pm and NiHC-4,4-bp at 1.47 V *vs*. RHE in 1 M KOH + 0.1 M substrates (substrates=benzyl alcohol, 1-phenylethanol, 3-phenyl-1-propanol and 1-phenyl-2-propanol). **c** Schematic illustration of the substrate configurations on NiHC-pm and NiHC-4,4-bp with 100% vacancy channels and 66.7% vacancy channels.

A key feature of the enzymes that has not been widely achieved on heterogeneous catalysis is the substrate selectivity owing to the specific protein pocket sizes^60^. The tunability of the ligand in the pillared inorganic-organic hybrids could potentially create the possibility of forming vacancy channels with different sizes, thereby mimicking the enzymatic properties. Herein, we utilized pyrimidine and 4,4-bipyridine as the N-N ligands, and successfully synthesized the structural analogs to our NiHC-pz (named as NiHC-pm and NiHC-4,4-bp). The XRD patterns of NiHC-pm pointed to slightly different structures with slightly distorted structure to realize the Ni-pyrimidine coordination and a shrunk channel size of ~6.2 Å × 9.7 Å (Fig. 5a, c). The NiHC-4,4-bp shares similarity to NiHC-pz but with elongated unit cell of 11.5 Å along the b direction (Fig. 5c). Employing the ultrahigh vacuum annealing to these alternatives could also create the active vacancy channels for obtaining similar activity trends as the NiHC-pz, demonstrating a general approach (Supplementary Figs. 38, 39).

More importantly, the different N-N ligands could generate the differently-sized vacancy channels to mimic the substrate-selectivity in enzymes. We compared the electrocatalytic oxidation activities of benzyl alcohol, 1-phenylethanol, 3-phenyl-1-propanol and 1-phenyl-2-propanol on NiHC-pm-250 and NiHC-4,4-bp-350, and observed different substrate dependence. The NiHC-pm-250 catalyst exhibited a high activity of 260 mA/mg for benzyl alcohol oxidation, but significantly decreased activities of 107 mA/mg, 89 mA/mg and 61 mA/mg when the substrates were enlarged to 1-phenylethanol, 3-phenyl-1-propanol and 1-phenyl-2-propanol respectively (Fig. 5b and Supplementary Fig. 40). Comparatively, the NiHC-4,4-bp-350 catalyst showed a slightly inferior activity of 249 mA/mg for benzyl alcohol, but the activity only subtly decayed to 200 mA/mg and 166 mA/mg for 1-phenylethanol and 3-phenyl-1-propanol, greatly outperforming NiHC-pm-250. Only when the substrate was 1-phenyl-2-propanol with a three-dimensionally larger size, the NiHC-4,4-bp-350 produced a drastically lower activity of 27 mA/mg. The isomers of 3-phenyl-1-propanol and 1-phenyl-2-propanol exhibited a significant 6.05 times difference in activity on NiHC-4,4-bp.

We further simulated the substrate configurations of NiHC-pm and NiHC-4,4-bp with different degrees of vacancy sites (Fig. 5c). We utilized a 3 Ni × 2 Ni × 4 Ni slab (NiHC-pm) or a 3 Ni × 2 Ni × 2 Ni slab (NiHC-4,4-bp) and removed a row of three ligands for simulating the vacancy channels (Supplementary Figs. 41, 42). Since the chlorides were discovered to be gradually substituted by the hydroxides during the catalysis (Supplementary Fig. 36), we utilized pure Ni hydroxide chains to mimic the intrinsic catalytically active site. The structures with pure Ni hydroxide chains exhibited highly consistent structures compared to the NiHC-pm-Cl or NiHC-4,4-bp-Cl except replacing all Cl ligands by the hydroxide ligands (Supplementary Figs. 43, 44). To identify the most stable configuration, we generate 10 adsorbed random conformations for each alcohol in the cavity using the distance geometry method^61^. (Fig. 5c and Supplementary Figs. 45–47) The optimized configurations on NiHC-pm all adopted a horizontally-inserted manner due to the small-sized channel, while these configurations exhibited more flexibilities along the b axis on NiHC-4,4-bp (Fig. 5c). Since full ligand removal was shown to cast adverse effects on the activities, we also simulated the configurations with lower degrees of cavity sites by only removing two-thirds of the ligands. The optimized configuration showed a crowdedly-filled vacancy site in NiHC-pm, while the larger empty spaces along the b axis allowed the substrate molecules to stretch along this direction to possess advantageous oxidation of larger substrates, which correlates well with the experimental results with substrate-dependent activities (Fig. 5c). Similar configuration results could be also be obtained on the NiHC-pm-Cl or NiHC-4,4-bp-Cl, pointing to a general approach of tuning the cavities via ligand sizes (Supplementary Figs. 48–51).

## Discussion

In this study, we discovered the creation of ligand vacancy channels of the NiHC-based hybrids could offer significant activity enhancement for the electrochemical oxidation of organics. The incorporation of hydroxide into the hybrid enhanced the structural integrity and allowed for the evacuation of the organic ligand via ultrahigh vacuum annealing. The removed ligands generated vacancy channels with a high density of undercoordinated Ni sites while the remaining ligands maintained the structural robustness. These vacancy channels not only create facile accessibility to the undercoordinated Ni sites, but also serve as entry points to dynamically exchange with the remaining ligand pillars for full utilization of the Ni sites. The typical NiHC-pz-300 could produce 5–25 fold and 20–400 fold activity enhancement compared to the parent NiHC-pz without vacancy channels and the bulk heterogeneous Ni(OH)_2_. The tunability of the pillared ligand enables the well-tailored sizes of the vacancy channels, creating new properties of the shape-dependent oxidation activities often pertaining to enzymes. Such a high level of tunability and generated new properties offer a potential future for efficient and smart catalysis.

## Methods

### Chemicals and materials

All reagents were used as received without further purification. Nickel chloride hexahydrate (NiCl_2_·6H_2_O), pyrazine, pyrimidine, 4,4-bipyridine, ethanol (≥ 99.7%), N,N-dimethylformamide, methanol, 2,2,2-trifluoroethanol, 2-phenylethanol, 1-propanol, 3-propanol, furfural, 1,4-butanediol, 1,6-hexanediol, ethylamine, 1-phenylethylamine, cyclohexanol, cyclohexylamine, 1-propylamine, 2-aminoethanol, urea, glycerol, glucose, 5-hydroxymethylfurfural (5-HMF), ethylene glycol and 1-hexanethiol were all purchased from Aladdin Industrial Corporation (China). Potassium hydroxide (KOH) was purchased from Sinopharm Chemical Reagent Co., Ltd. (China). 1,1,1-trifluoro-2-propanol, 1-phenylethanol, benzaldehyde and 2-propylamine were purchased from Innochem Alfa Acros (China). Methyl mercaptan was purchased from Macklin Biochemical Co., Ltd. (China). 2,2,2-trifluoroethylamine and benzylamine were purchased from Shanghai Titan Scientific Co., Ltd. (China). Ultrapure deionized water (18.2 MΩ·cm^−1^, 25 ^o^C) was obtained from ELGA purification system (China). Anion exchange membrane was obtained from Fumatech (FAB-PK-130, Germany). Carbon fiber paper was purchased from Hesen Electric Co., Ltd. (HCP020N, China).

### Catalyst preparation

For the NiHC-pz (pz = pyrazine) synthesis, 0.96 mmol NiCl_2_·6H_2_O was completely resolved in 12 mL DMF and 1.2 mL water. Then, 0.96 mmol pyrazine was added to the mixed solution and reacted at 120 °C for 24 h to obtain the final NiHC-pz electrocatalyst. For NiHC-pm (pm = pyrimidine) and NiHC-4,4-bp (4,4-bp = 4,4-bipyridine), the preparation methods were similar to that of the NiHC-pz by just substituting the ligands from pyrazine to pyrimidine and 4,4-bipyridine. The as-synthesized powders were annealed at various temperature for 1 h under an ultrahigh vacuum atmosphere (10^−3^ Pa, ramping rate of 5 °C min^−1^). The materials were denoted as NiHC-ligand-temperature.

### Thiol poisoned catalyst preparation

0.2 g NiHC-pz-300 catalyst, 10 mL DMF and 1 mL vulcanizing reagent (methyl mercaptan or n-hexyl mercaptan) were mixed well and aged for 24 h at 40 °C. After this progress, the products were centrifuged and washed with ethanol several times, and then dried at 60 °C. The as-treated samples were directly used for subsequent FTIR and electrochemical tests.

### Materials characterization

The crystal structure was characterized by a Bruker D8 Advance powder X-ray diffractometer (PXRD) equipped with a Cu Kα X-ray source (λ = 1.5418 Å) and a Lynxeye detector. PXRD scans were collected within the 2θ range of 5° to 90° with a step size of 0.02°. TEM images were acquired on a FEI Tecnai G2 F20 S-Twin equipped with operated at 200 kV. The elemental composition was obtained by X-ray photoelectron spectroscopy (XPS, Thermo Scientific K-Alpha) with Al Kα radiation source (hν = 1486.6 eV). The position of the C 1 s peak at 284.8 eV was employed to be a calibration reference to determine the accurate binding energies (± 0.1 eV). Attenuated total reflection Fourier transform infrared absorption spectroscopy (ATR-FTIR) were acquired at a resolution of 4 cm^−1^ with unpolarized IR radiation at an incidence angle of ca. 70° by a Nicolet iS20 FTIR spectrometer (Nicolet iS20 FTIR, Thermo Scientific) with a built-in MCT detector. All spectra were converted to the absorbance unit as -log (I/I_0_), where I and I_0_ represent the intensities of the reflected radiation of the sample and reference spectra, respectively. The Ni K-edge X-ray adsorption spectroscopy (XAS) were performed at the 1W1B beamline of the Beijing Synchrotron Radiation Facility (BSRF). The TG-FTIR-GCMS analysis was performed on a TGA8000-Frontier FTIR-SQ8 GCMS from Perkin Elmer. The TGA adopted a ramping rate of 20 K/min from 35 ^o^C to 800 ^o^C in high-purity nitrogen The N_2_ blow rate was 60 mL/min, the pumping rate was set at 45 mL/min, and the tubing was heated at 270 ^o^C. The FTIR utilized the resolution of 4 cm^−1^ with 4 collection cycles each temperature point. The FTIR spectra were collected at 270 ^o^C. The MS utilized the ion flow mode with the mass of 17, 18, 36, 38, 53, and 80 amu. High purity He was utilized as the carrier gas with the inlet heated at 250^o^C. The FTIR and MS were adjusted to the same time and constantly monitored for the time.

### Electrochemical measurements

The as-prepared NiHC-pz-300 catalyst (12.5 mg) was dispersed in 968 μL absolute ethanol and 32 μL Nafion solution (5 wt%) accompanied by a continuous ultra-sonification to form a homogeneous catalyst ink. Then, 40 μL ink was pipetted onto the double sides of carbon paper, giving a catalyst loading of 1 mg cm^−2^. The catalyst with lower loadings was prepared by diluting the ink with ethanol. The electrochemical workstation (CHI 660E, Shanghai CH Instruments Co., China) was utilized for the electrochemical studies. The electrochemical measurements were carried out in a typical H-Type cell with three-electrode configuration, which consists of the as-prepared NiHC-pz-300 catalyst electrode as the working electrode, a platinum foil as the auxiliary electrode, and a Ag/AgCl (saturated KCl) as the reference electrode. All measured potentials were converted to the reversible hydrogen electrode (RHE) according to the following equation:

1 $${{{{{\rm{E}}}}}}({{{{{\rm{RHE}}}}}})={{{{{\rm{E}}}}}}({{{{{\rm{Ag}}}}}}/{{{{{\rm{AgCl}}}}}})+0.197+0.0591\times {{{{{\rm{pH}}}}}}$$

The electrochemical oxidation activity of 25 organic substrates (methanol, ethanol, 2,2,2-trifluoroethanol, benzyl alcohol, 2-propanol, 1,1,1-trifluoro-2-propanol, 1-phenylethanol, benzaldehyde, furfural, ethylene glycol, 1,4-butanediol, 1,6-hexanediol, ethylamine, 1-propylamine, 2,2,2-trifluoroethylamine, benzylamine, 2-propylamine, 1-phenylethylamine, cyclohexanol, cyclohexylamine, urea, glycerol, glucose, 5-hydroxymethylfurfural and 2-aminoethanol) were evaluated in 1 M KOH + 0.1 M substrate. The Linear sweep voltammetry (LSV) curves were scanned at a rate of 5 mV s^−1^ at room temperature after 5 cyclic voltammetry (CV) cycles at a scan rate of 50 mV s^−1^. All polarization curves were manually corrected with 90% iR-compensation. For obtaining accurate Tafel slope values, all Tafel plots were iR-corrected. Chronopotentiometric measurements were recorded at a current density of 20 mA cm^−2^. In order to reduce the impact on the stability of the catalyst due to the changes of substrate concentration, the electrolyte was refreshed every 12 h. Turnover frequencies (TOFs) were calculated from the following equation:

2 $${{{{{\rm{TOF}}}}}}=\frac{I}{{nFc}}\,$$

where *I* is the current density in the LSV curve (mA/mg), *n* is the number electrons needed for the oxidation of one urea molecule (*n* = 6 (N_2_) or 12 (NO_2_^−^)), *F* is the Faraday constant of 96485 F/mol, *c* is the active Ni site density in the catalyst (mol/g).

### In situ Raman measurements

In situ Raman spectra were collected using Horiba Jobin Yvon, equipped with a 532 nm laser and a 2400 mm^−1^ grating. The resolution of this Raman spectrometer was about 1.3 cm^−1^ and the acquisition time was 50 s. All spectra were measured from 200 to 1800 cm^−1^. The in situ Raman spectroscopy was conducted on a three-electrode cell (EC-RAIR, Beijing Science Star Technology Co., Ltd.) with a Pt wire as the counter electrode, Ag/AgCl electrode as the reference electrode and gold as the working electrode (catalyst loading: 10 µg). During measurement, a series of potentials were applied to the working electrode. The electrolytes of 1 M KOH + 0.1 M substrates were sealed in a capillary tube with an inner diameter of 1 mm for Raman measurements before and after the electrolysis.

### Ligand quantification

To determine the pyrazine content in NiHC-pz and its pyrolysis products, the nuclear magnetic resonance (NMR, Bruker 400 MHz Avance III HD) spectrometer was conducted. Before the NMR measurement, all samples were immersed in aqua regia for 24 h. After the solids dissolved completely, sufficient sodium hydroxide solution was added slowly to the above solution until no precipitation occurred. Then, transparent solution contained pyrazine molecules was acquired after filtering with inorganic membrane. For NMR measurements, 100 μL as-prepared solution was added into a 500 μL D_2_O solution with 10 µmol DMSO as the internal standard.

### Computational methods

The crystal structures of NiHC-pm and NiHC-4,4-bp supercells used in this work consist of 24 NiO_2_H_2_ + 24 C_4_H_4_N_2_ and 12 NiO_2_H_2_ + 12 C_10_H_8_N_2_ with dimensions of 16.01 Å × 24.10 Å × 9.84 Å and 12.24 Å × 22.50 Å × 10.23 Å, respectively. Then, one C_4_H_6_N_2_ or C_10_H_8_N_2_ molecule in the framework is deleted to form the cavities in the NiHC-pm and NiHC-4,4-bp. The coordinates of the final structure are given in the supporting information. All calculations are performed using Vienna Ab initio Simulation (VASP)^62^ with the projector augmented wave (PAW) potentials and the Hubbard term corrected DFT functional, PBE + U^63,64^. The effective Hubbard term (U_eff_) is set 5.5 eV for Ni in accordance with the linear response approach^65–67^. The kinetic cutoff energy is 500 eV. Owing to the large size of the surface cell, the Γ-point sampling is utilized for the NiHC-pm and NiHC-4,4-bp. The Quasi-Newton L-BFGS method is used for geometry relaxation until the maximal force on each degree of freedom is less than 0.08 eV/Å.

### Reporting summary

Further information on research design is available in the Nature Portfolio Reporting Summary linked to this article.

## Supplementary information

Supplementary Information

Dataset 1

Reporting Summary

## Data Availability

The data that support the findings of this study are available from the corresponding author upon reasonable request.
