# Supplementary material for: Ligand vacancy channels in pillared inorganic-organic hybrids for electrocatalytic organic oxidation with enzyme-like activities
Source: Nat Commun. 2023 Mar 2;14:1184. doi: 10.1038/s41467-023-36830-4 (PMC9981682; doi:10.1038/s41467-023-36830-4)
Supplement: Supplementary file 1 — Supplementary Information [file 41467_2023_36830_MOESM1_ESM.pdf]

Supplementary Information

Ligand Vacancy Channels in Pillared Inorganic-Organic Hybrids for Electrocatalytic Organic Oxidation with Enzyme-like Activities

Zhe Chen[1‡], Jili Li[1‡], Lingshen Meng[1], Jianan Li[2], Yaming Hao[1], Tao Jiang[1], Xuejing Yang[2], Yefei Li[1*], Zhi-Pan Liu[1], Ming Gong[1*]

[1]Department of Chemistry and Shanghai Key Laboratory of Molecular Catalysis and Innovative Materials, Fudan University, Shanghai, China, 200438

[2] National Engineering Laboratory for Industrial Wastewater Treatment, East China University of Science and Technology, Shanghai, China, 200237

[‡]These authors contributed equally to this work.

To whom correspondence may be addressed. Email: gongm@fudan.edu.cn; yefeil@fudan.edu.cn

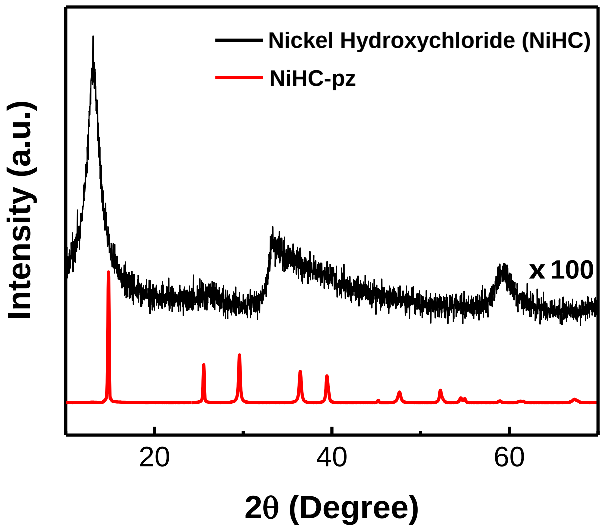

Supplementary Fig. 1. The PXRD pattern of the NiHC and NiHC-pz.

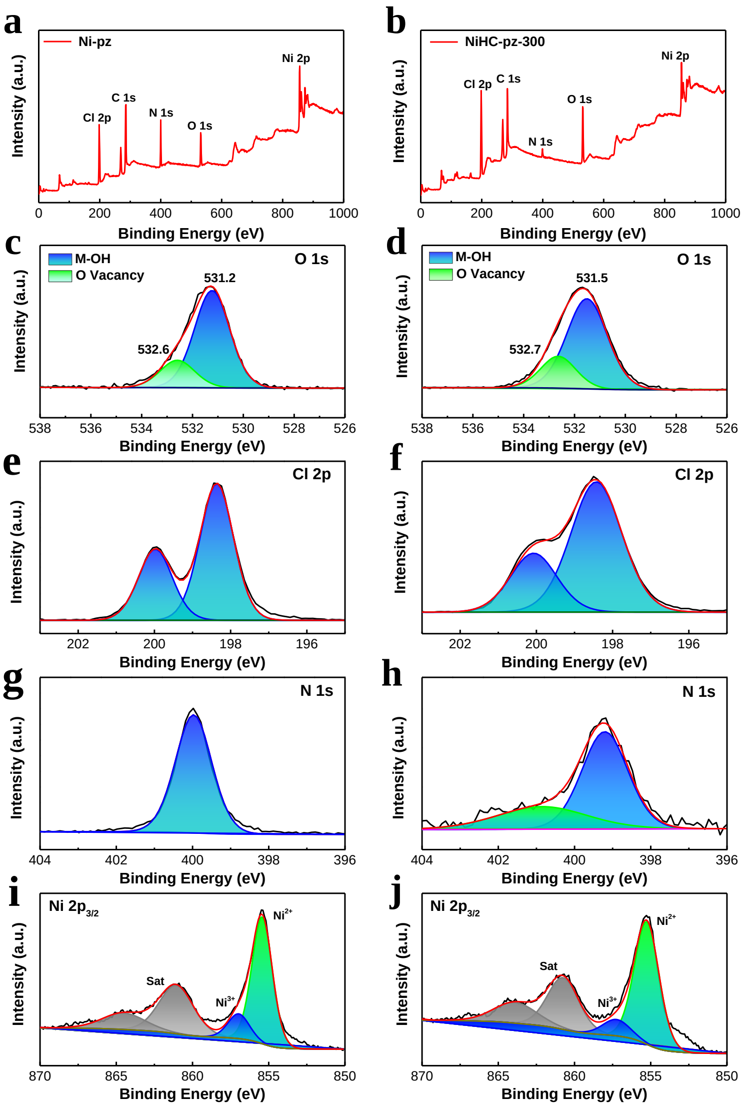

Supplementary Fig. 2. The X-ray photoelectron spectra (XPS) of the NiHC-pz (a) and NiHC-pz-300 (b). The XPS spectra of (c) O 1s, (e) Cl 2p, (g) N 1s, and (i) Ni $2p_{3/2}$ in NiHC-pz. The XPS spectra of (d) O 1s, (f) Cl 2p, (h) N 1s, and (j) Ni $2p_{3/2}$ in NiHC-pz-300. After the removal of pyrazine, the Ni^{+2} peak was slightly broadened compared to the pristine material, which indicated an increased heterogeneity with local coordination environment changes. These changes also affected the local coordination environment of chloride and hydroxide by showing the broadened Cl 2p peak and shifted M-OH peak toward larger O 1s binding energy in XPS. The N spectrum of NiHC-pz-300 shows the significant loss of N signals, as well as the shift of remaining N toward lower binding energy, presumably due to the weakening coordination bond of the remaining pyrazine with Ni.

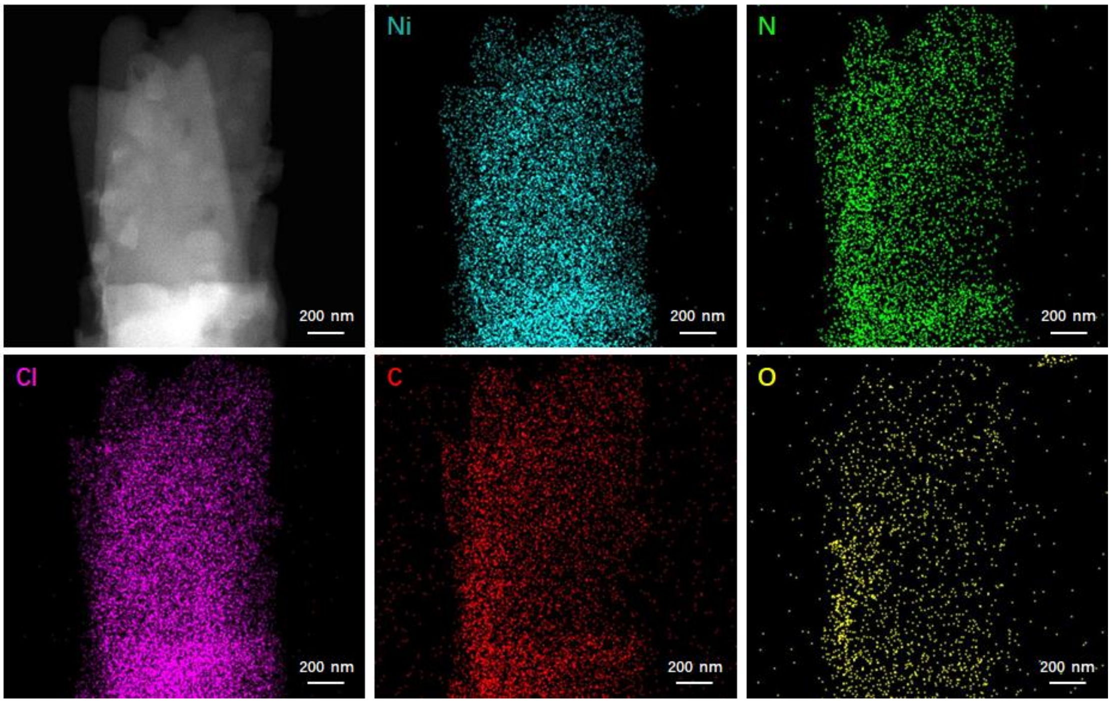

Supplementary Fig. 3. The TEM image of NiHC-pz and relevant EDS elemental maps of C, N, O, Cl, and Ni.

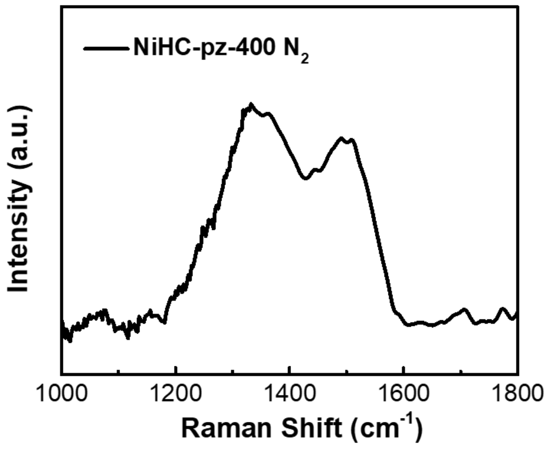

Supplementary Fig. 4. The Raman spectrum of NiHC-pz-400 N_2.

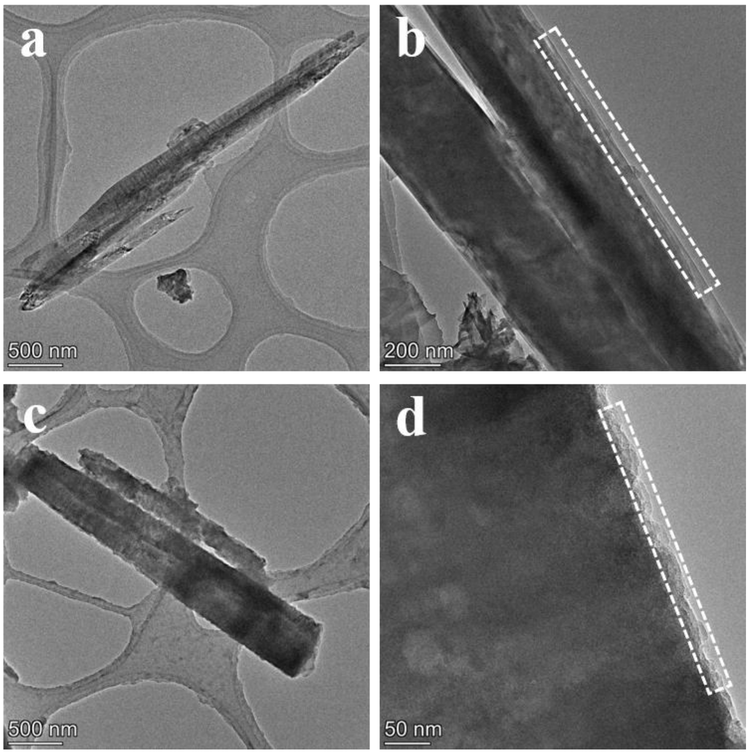

Supplementary Fig. 5. (a, b) The TEM images of NiHC-pz. (c, d) The TEM images of NiHC-pz-300.

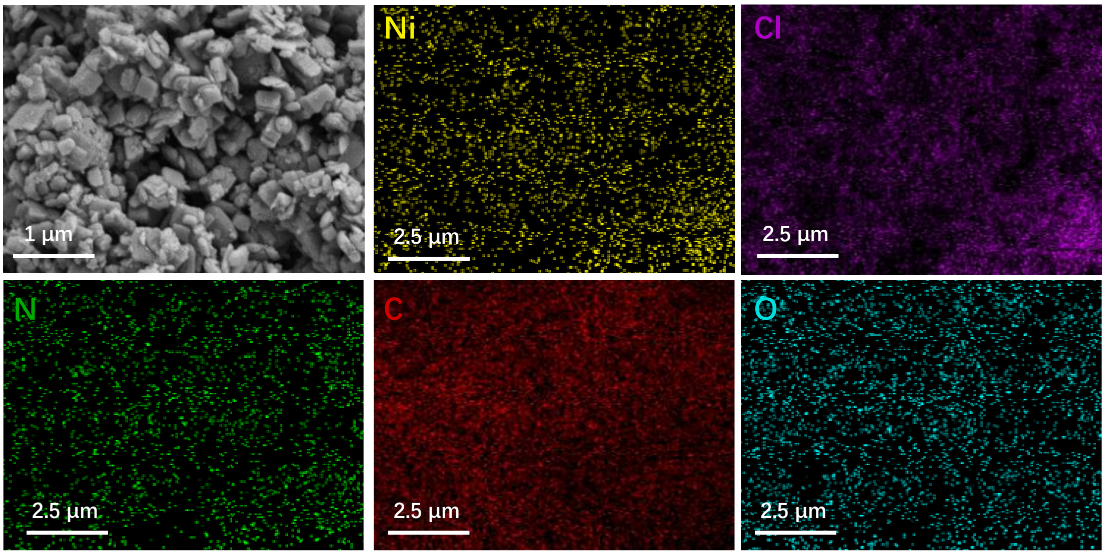

Supplementary Fig. 6. The SEM image of $NiCl_2(pz)$ and relevant EDS elemental maps of C, N, O, and Ni.

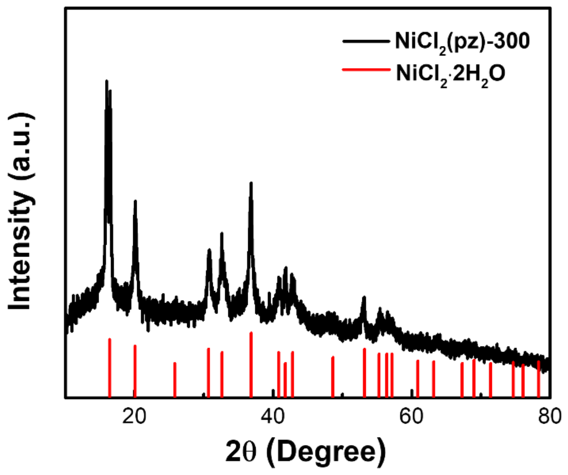

Supplementary Fig. 7. The PXRD pattern of $NiCl_2$(pz)-300.

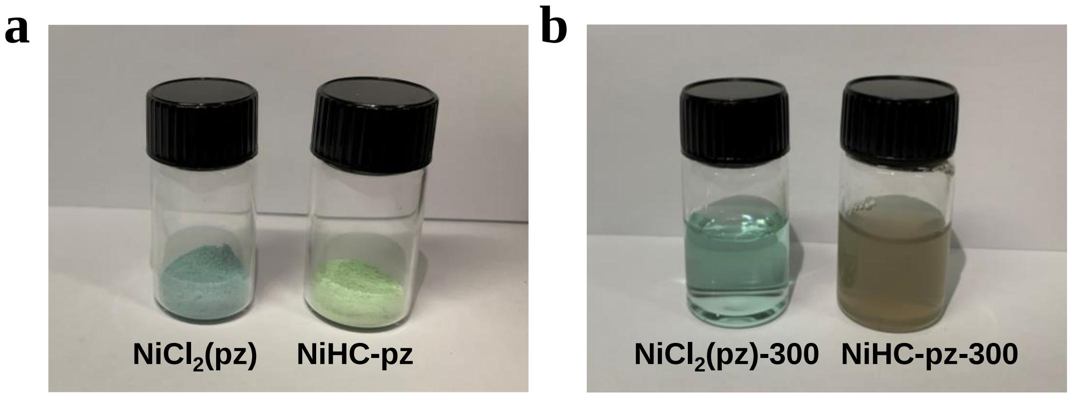

Supplementary Fig. 8. (a) Photos of NiHC-pz and $NiCl_2$(pz); (b) Photos of NiHC-pz-300 and $NiCl_2$(pz)-300 suspended or dissolved in water.

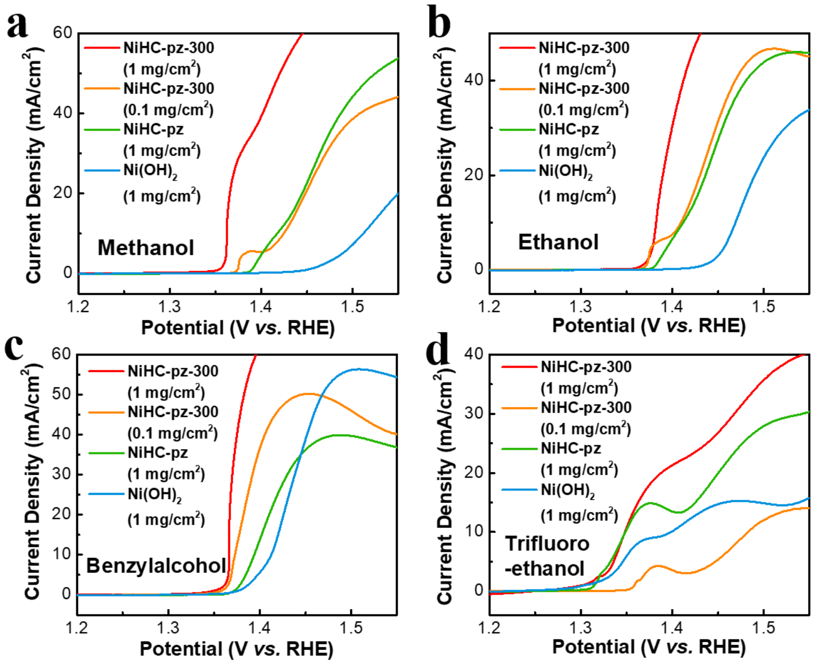

Supplementary Fig. 9. The iR compensated polarization curves of NiHC-pz-300, NiHC-pz-300 (10%, catalyst loading 0.1 mg cm^{-2}), NiHC-pz and $Ni(OH)_2$ in 1 M KOH and 0.1 M (a) methanol, (b) ethanol, (c) benzyl alcohol, and (d) 2,2,2-trifluoroethanol at a scan rate of 5 mV s^{-1}.

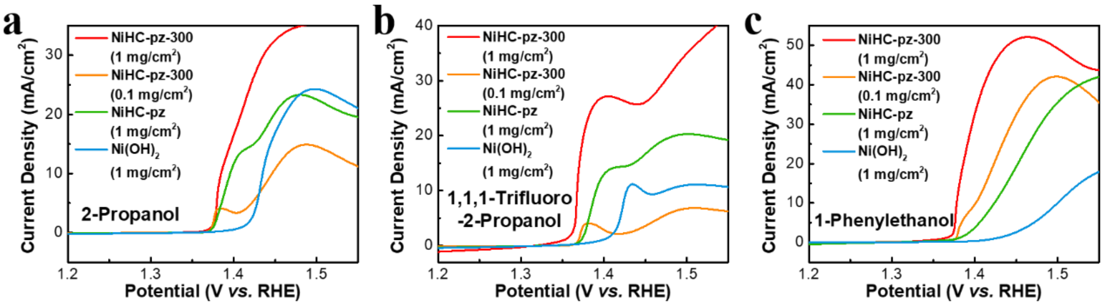

Supplementary Fig. 10. The iR compensated polarization curves of NiHC-pz-300, NiHC-pz-300 (10%), NiHC-pz and $Ni(OH)_2$ in 1 M KOH and 0.1 M (a) 2-propanol, (b)1,1,1-trifluoro-2-propanol, and (c) 1-phenylethanol at a scan rate of 5 mV s^{-1}.

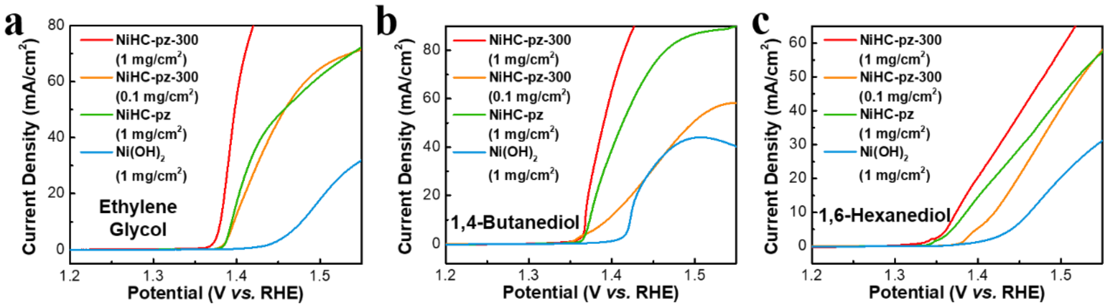

Supplementary Fig. 11. The iR compensated polarization curves of NiHC-pz-300, NiHC-pz-300 (10%), NiHC-pz and $Ni(OH)_2$ in 1 M KOH and 0.1 M (a) ethylene glycol, (b) 1,4-butanediol, and (c) 1,6-hexanediol at a scan rate of 5 mV s^{-1}.

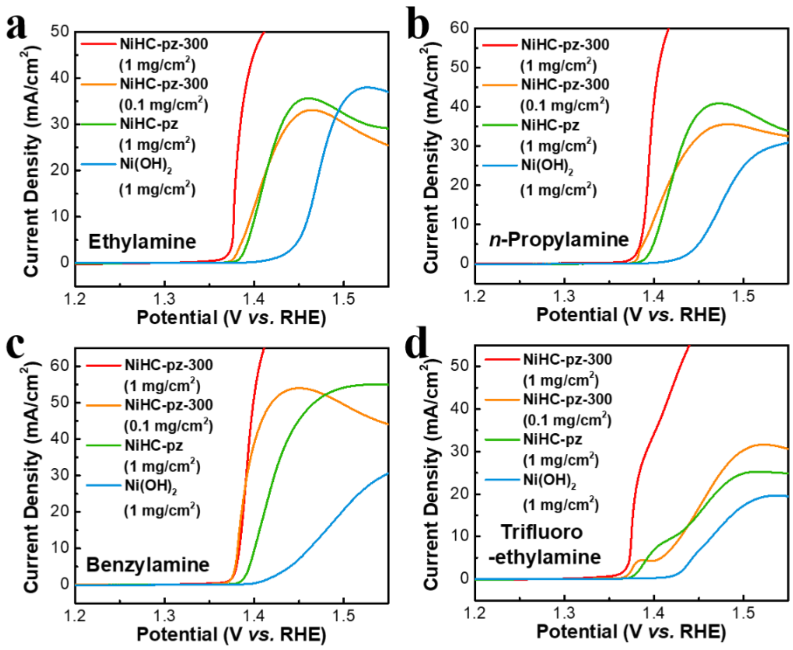

Supplementary Fig. 12. The iR compensated polarization curves of NiHC-pz-300, NiHC-pz-300 (10%), NiHC-pz and $Ni(OH)_2$ in 1 M KOH and 0.1 M (a) ethylamine, (b) *n*-propylamine, (c) benzylamine, and (d) trifluoroethylamine at a scan rate of 5 mV s^{-1}.

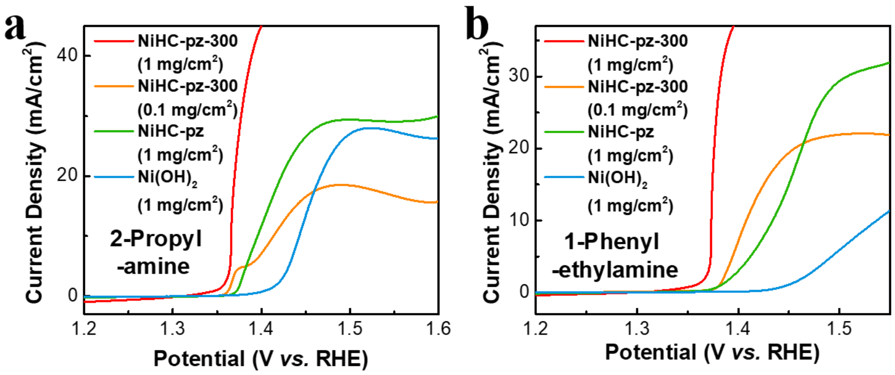

Supplementary Fig. 13. The iR compensated polarization curves of NiHC-pz-300, NiHC-pz-300 (10%), NiHC-pz and $Ni(OH)_2$ in 1 M KOH and 0.1 M (a) 2-propylamine, and (b) 1-phenylethylamine at a scan rate of 5 mV s^{-1}.

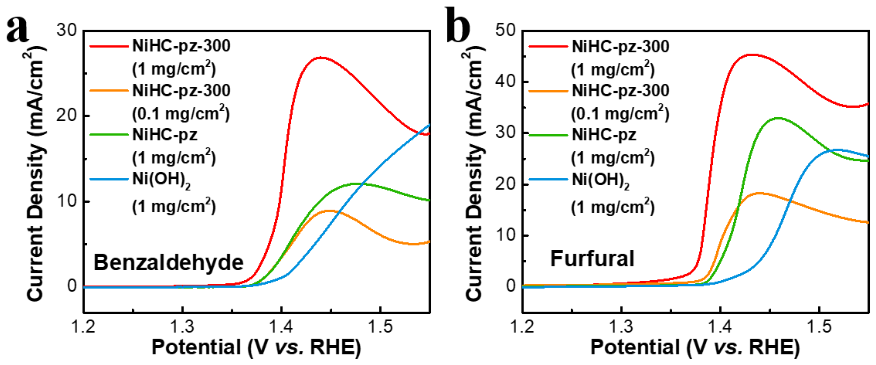

Supplementary Fig. 14. The iR compensated polarization curves of NiHC-pz-300, NiHC-pz-300 (10%), NiHC-pz and $Ni(OH)_2$ in 1 M KOH and 0.1 M (a) benzaldehyde, and (b) furfural at a scan rate of 5 mV s^{-1}.

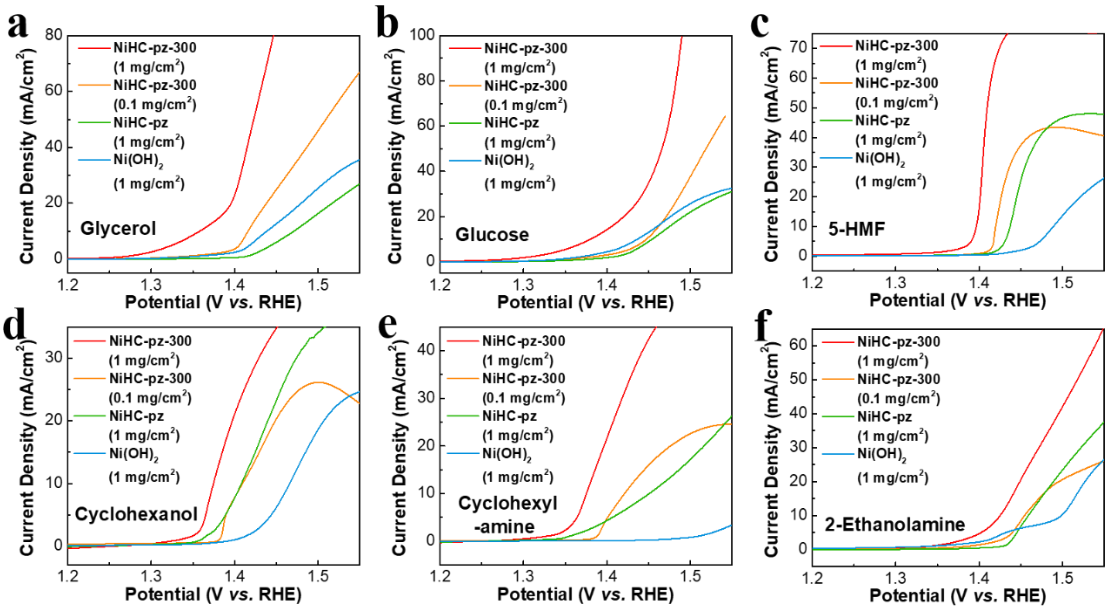

Supplementary Fig. 15. The iR compensated polarization curves of NiHC-pz-300, NiHC-pz-300 (10%), NiHC-pz and $Ni(OH)_2$ in 1 M KOH and 0.1 M (a) glycerol, (b) glucose, (c) 5-HMF, (d) cyclohexanol, (e) cyclohexylamine, and (f) 2-ethanolamine at a scan rate of 5 mV s^{-1}.

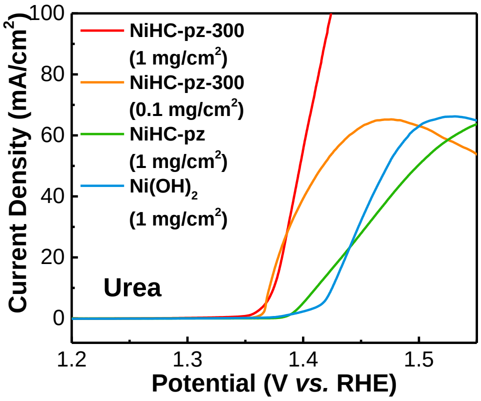

Supplementary Fig. 16. The iR compensated polarization curves of NiHC-pz-300, NiHC-pz-300 (10%), NiHC-pz and $Ni(OH)_2$ in 1 M KOH and 0.1 M urea at a scan rate of 5 mV s^{-1}.

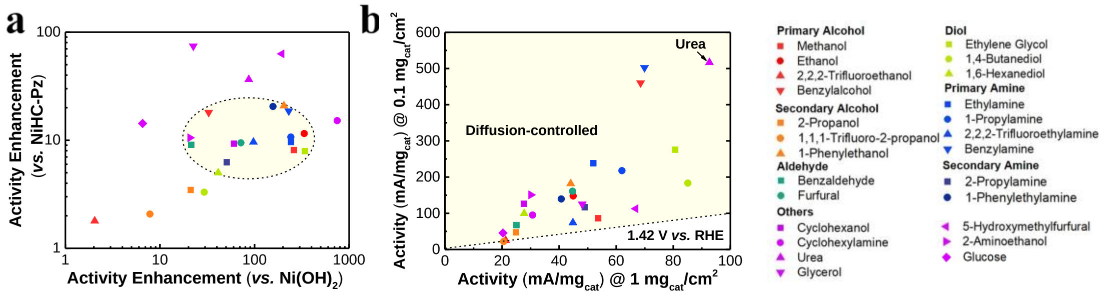

Supplementary Fig. 17. (a) The activity enhancement of NiHC-pz-300 compared to NiHC-pz and β-$Ni(OH)_2$ at 1.42 V *vs.* RHE for 25 different substrates (0.1 M substrate in 1 M KOH); (b) the gravimetric current densities of NiHC-pz-300 at the loadings of 0.1 mg_{cat}/cm^2 and 1 mg_{cat}/cm^2 for 25 different substrates showing diffusion-controlled kinetics.

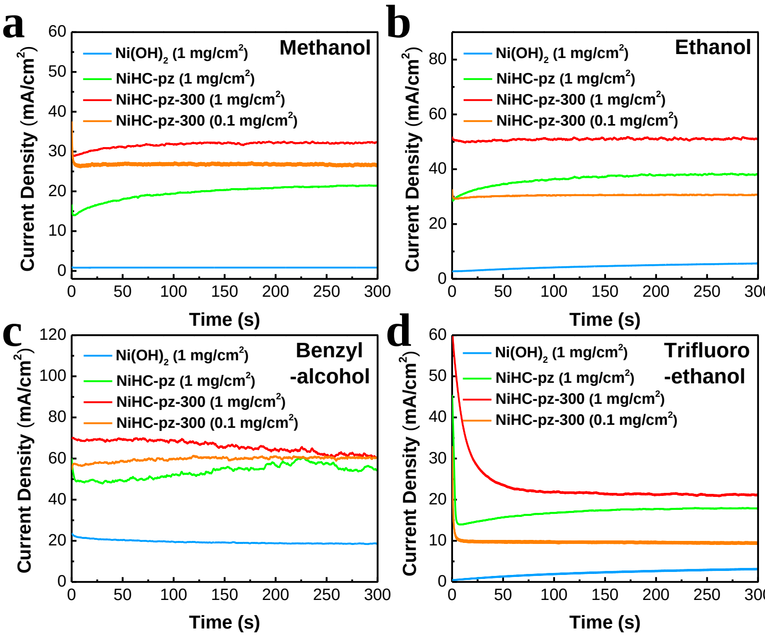

Supplementary Fig. 18. The chronoamperometry curves of NiHC-pz-300, NiHC-pz-300 (10%, catalyst loading 0.1 mg cm^{-2}), NiHC-pz and β-$Ni(OH)_2$ in 1 M KOH and 0.1 M (a) methanol, (b) ethanol, (c) benzyl alcohol, and (d) 2,2,2-trifluoroethanol at a potential of 1.45 V *vs*. RHE.

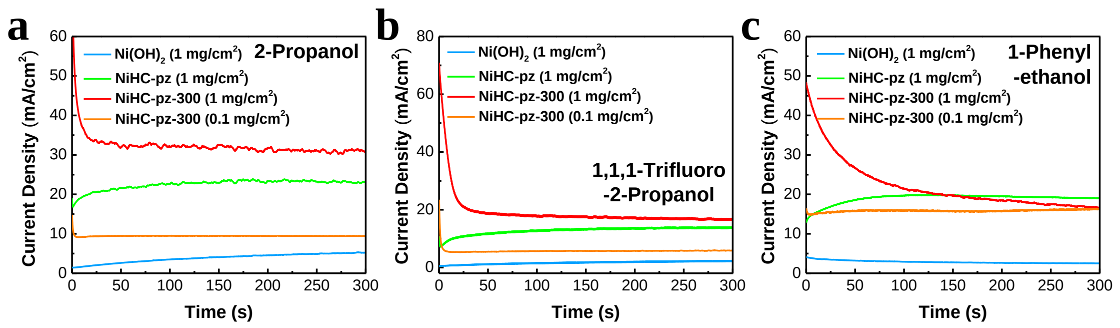

Supplementary Fig. 19. The chronoamperometry curves of NiHC-pz-300, NiHC-pz-300 (10%), NiHC-pz and β-$Ni(OH)_2$ in 1 M KOH and 0.1 M (a) 2-propanol, (b)1,1,1-trifluoro-2-propanol, and (c) 1-phenylethanol at a potential of 1.45 V *vs*. RHE.

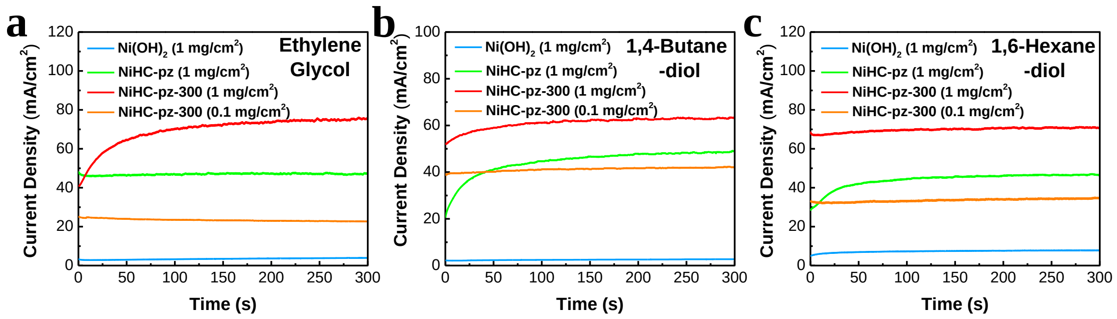

Supplementary Fig. 20. The chronoamperometry curves of NiHC-pz-300, NiHC-pz-300 (10%), NiHC-pz and β-$Ni(OH)_2$ in 1 M KOH and 0.1 M (a) ethylene glycol, (b) 1,4-butanediol, and (c) 1,6-hexanediol at a potential of 1.45 V *vs*. RHE.

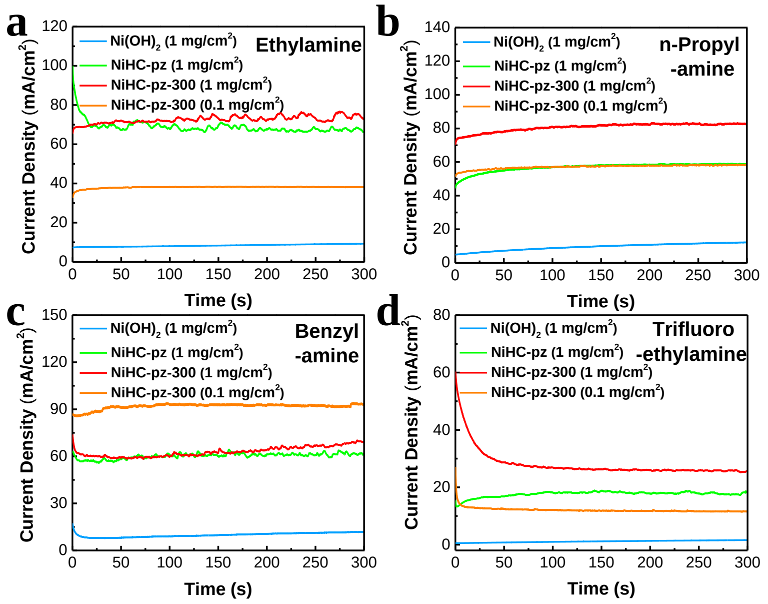

Supplementary Fig. 21. The chronoamperometry curves of NiHC-pz-300, NiHC-pz-300 (10%), NiHC-pz and β-$Ni(OH)_2$ in 1 M KOH and 0.1 M (a) ethylamine, (b) *n*-propylamine, (c) benzylamine, and (d) trifluoroethylamine at a potential of 1.45 V *vs*. RHE.

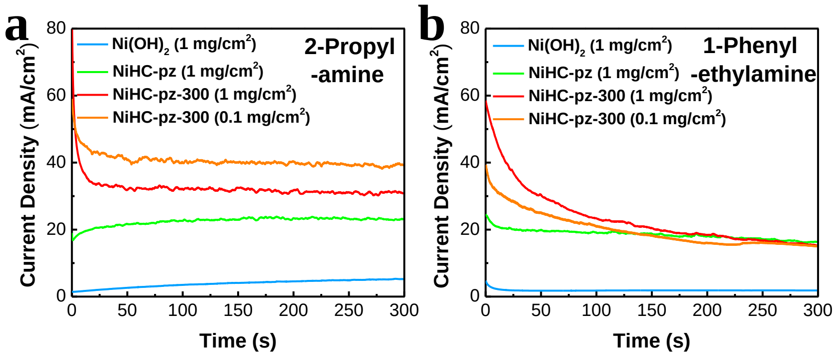

Supplementary Fig. 22. The chronoamperometry curves of NiHC-pz-300, NiHC-pz-300 (10%), NiHC-pz and β-$Ni(OH)_2$ in 1 M KOH and 0.1 M (a) 2-propylamine, and (b) 1-phenylethylamine at a potential of 1.45 V *vs*. RHE.

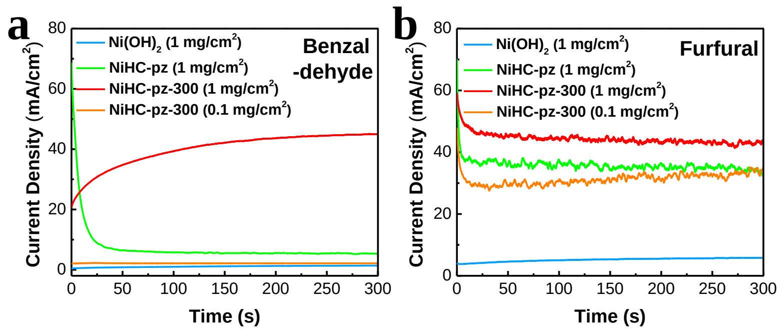

Supplementary Fig. 23. The chronoamperometry curves of NiHC-pz-300, NiHC-pz-300 (10%), NiHC-pz and β-$Ni(OH)_2$ in 1 M KOH and 0.1 M (a) benzaldehyde, and (b) furfural at a potential of 1.45 V *vs*. RHE.

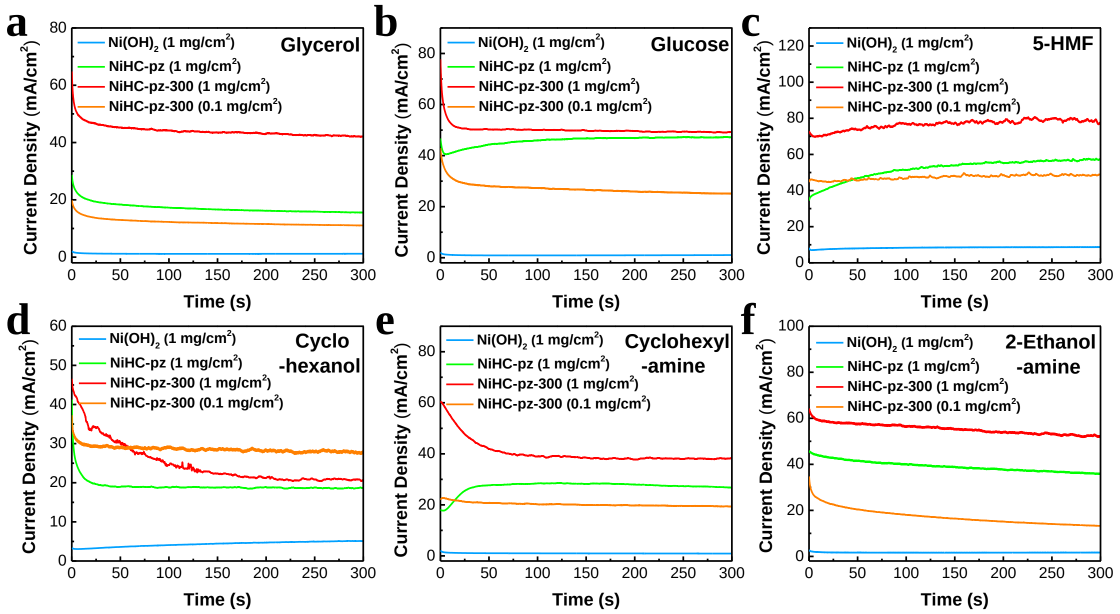

Supplementary Fig. 24. The chronoamperometry curves of NiHC-pz-300, NiHC-pz-300 (10%), NiHC-pz and β-$Ni(OH)_2$ in 1 M KOH and 0.1 M (a) glycerol, (b) glucose, (c) 5-HMF, (d) cyclohexanol, (e) cyclohexylamine, and (f) 2-ethanolamine at a potential of 1.45 V *vs*. RHE.

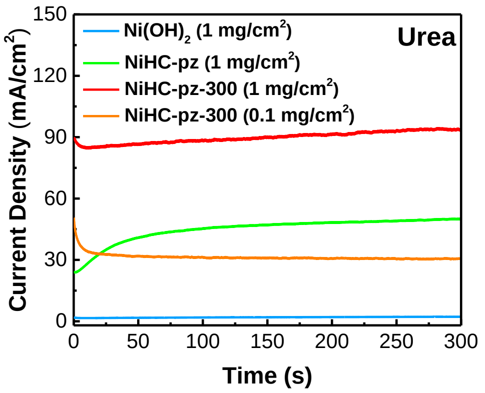

Supplementary Fig. 25. The chronoamperometry curves of NiHC-pz-300, NiHC-pz-300 (10%), NiHC-pz and β-$Ni(OH)_2$ in 1 M KOH and 0.1 M urea at a potential of 1.45 V *vs*. RHE.

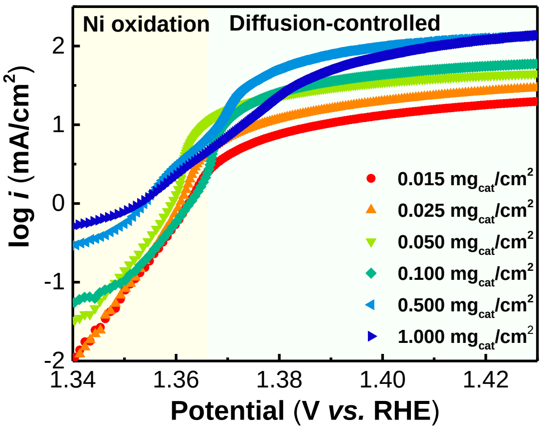

Supplementary Fig. 26. The Tafel plots of NiHC-pz-300 under different loadings in 1 M KOH + 0.1 M urea.

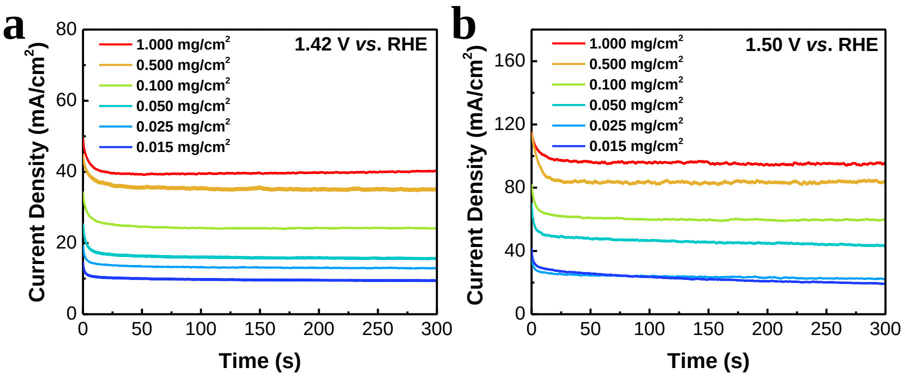

Supplementary Fig. 27. The chronoamperometry curves of NiHC-pz-300 at different loadings at 1.42 V *vs.* RHE and 1.50 V *vs.* RHE in 1 M KOH + 0.1 M urea.

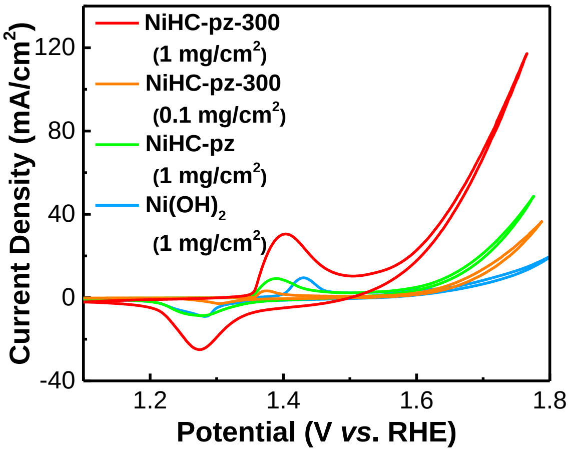

Supplementary Fig. 28. The CV curves of NiHC-pz-300, NiHC-pz-300 (10%), NiHC-pz and β-$Ni(OH)_2$ in 1 M KOH at a scan rate of 5 mV/s.

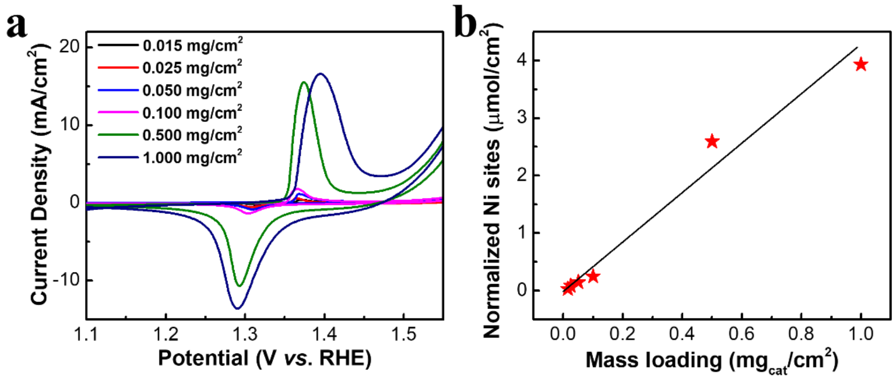

Supplementary Fig. 29. The CV curves and calculated Ni sites of NiHC-pz-300 in 1 M KOH at different loadings and a scan rate of 2 mV/s.

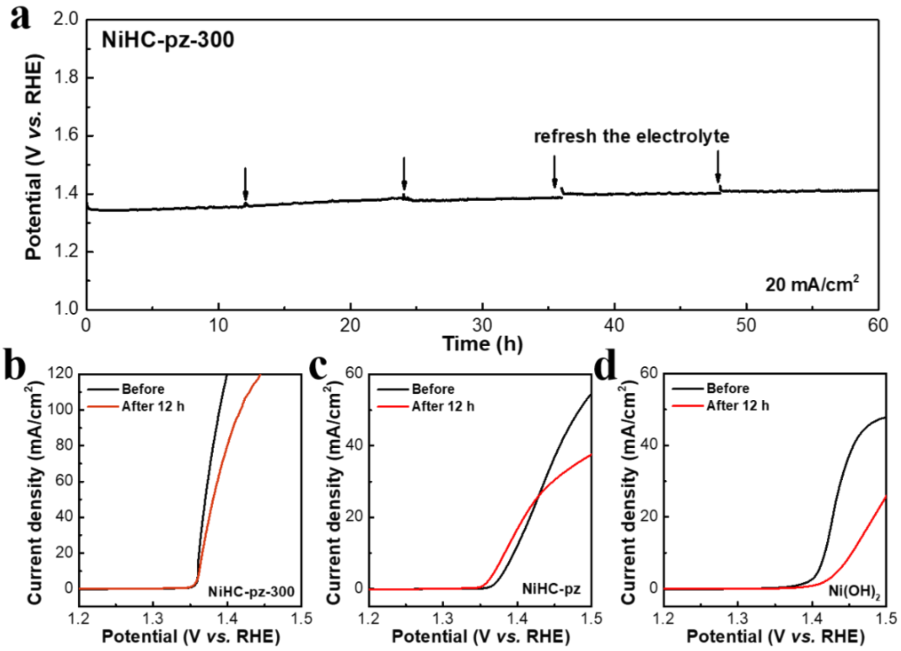

Supplementary Fig. 30. (a) The chronoamperometry curve of NiHC-pz-300 in 1 M KOH + 0.1 M urea, the electrolyte was refreshed every 12 hours. (b-d) The iR compensated polarization curves of NiHC-pz-300, NiHC-pz and $Ni(OH)_2$ in 1 M KOH and 0.1 M urea before and after 12 h electrolysis.

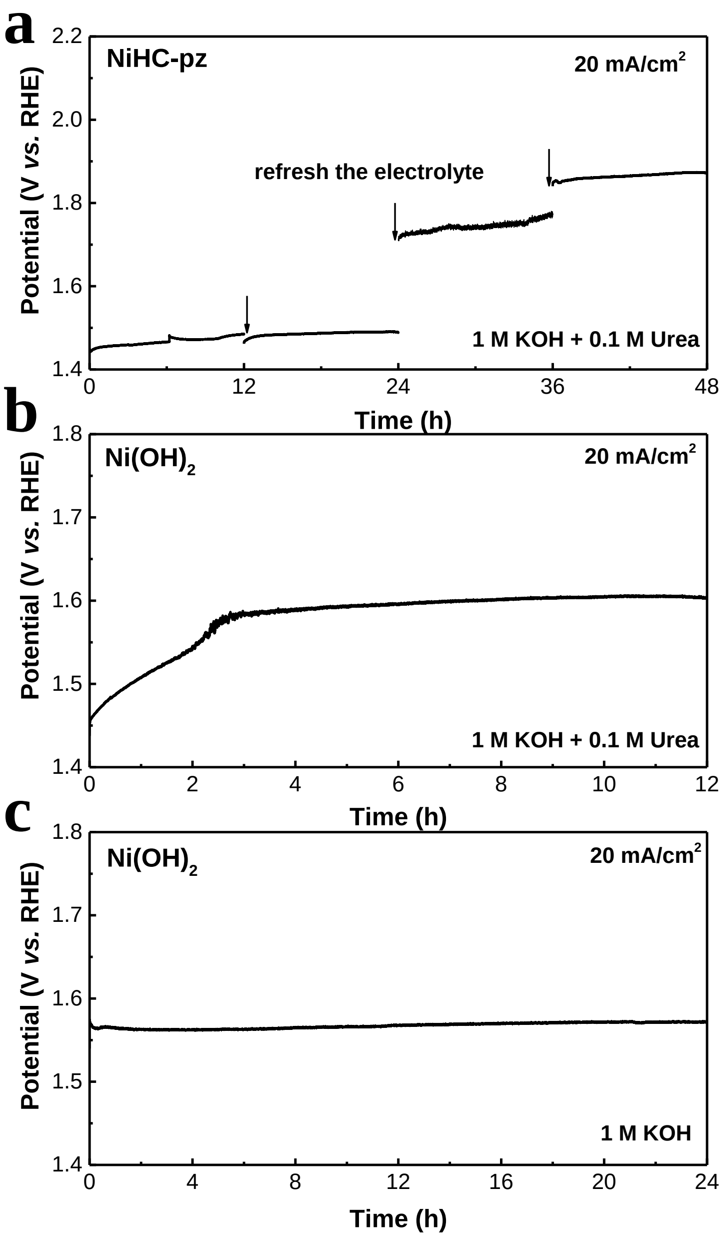

Supplementary Fig. 31. (a) The chronopotentiometry curve of NiHC-pz in 1 M KOH + 0.1 M urea, the electrolyte was refreshed every 12 hours. (b) The chronopotentiometry curve of β-$Ni(OH)_2$ in 1 M KOH + 0.1 M urea. (c) The chronopotentiometry curve of β-$Ni(OH)_2$ in 1 M KOH for oxygen evolution reaction.

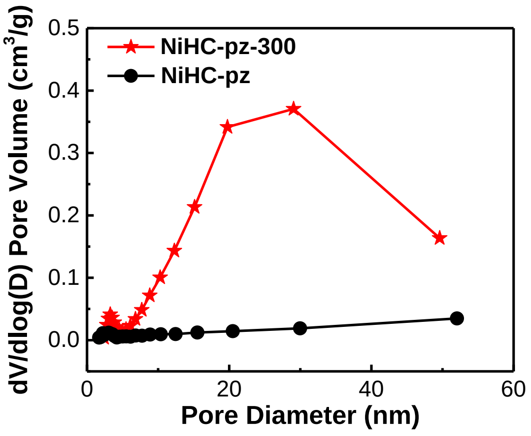

Supplementary Fig. 32. The pore size distribution of NiHC-pz and NiHC-pz-300.

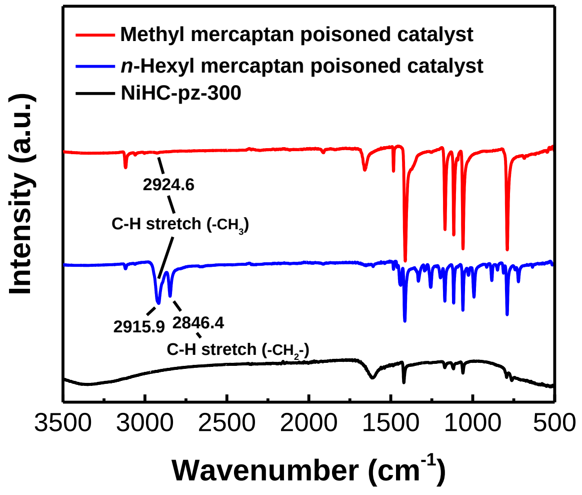

Supplementary Fig. 33. FT-IR spectra of NiHC-pz-300 poisoned by methyl mercaptan or hexyl mercaptan.

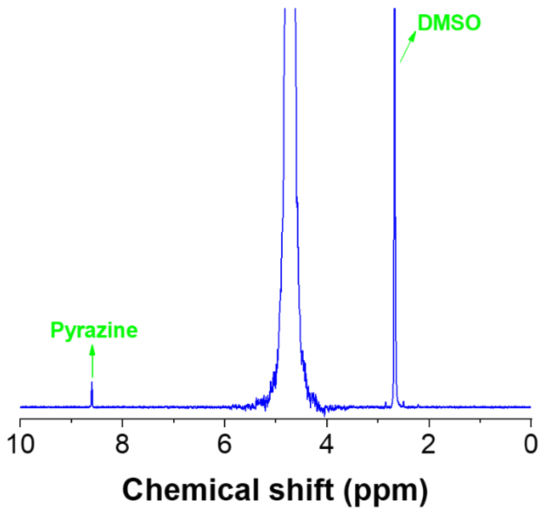

Supplementary Fig. 34. NMR spectrum of the pyrazine molecule in NiHC-pz-300.

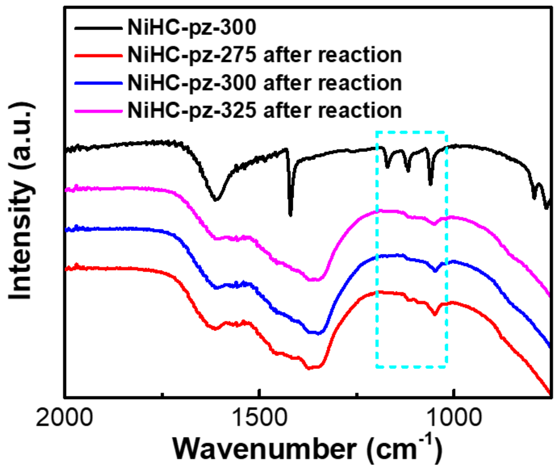

Supplementary Fig. 35. The FT-IR spectra of NiHC-pz-300 before and after the oxidation reaction, and NiHC-pz-275, NiHC-pz-325 after the reaction in 1 M KOH + 0.1 M urea.

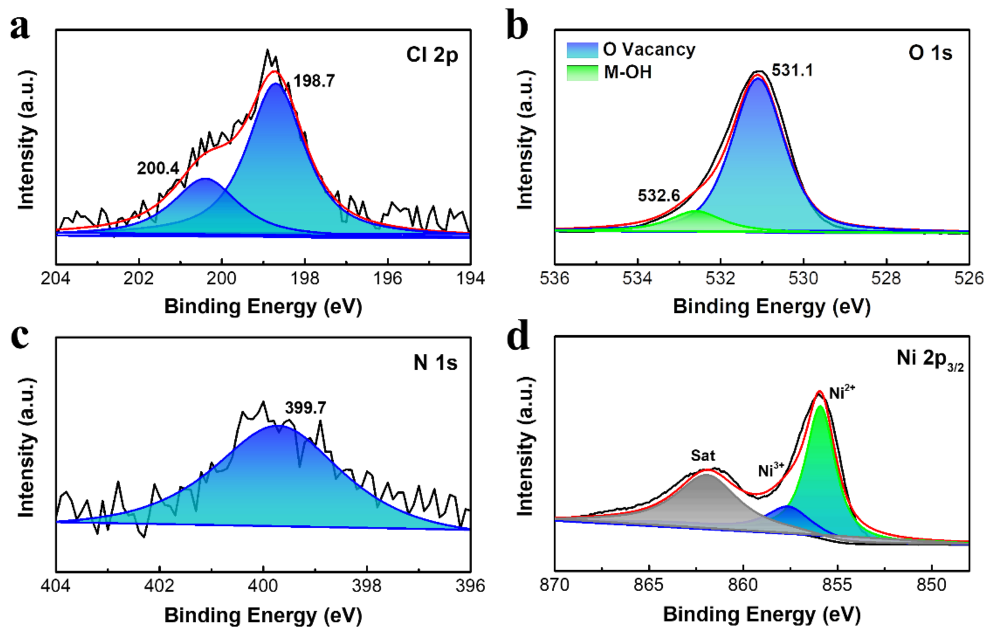

Supplementary Fig. 36. The XPS spectra of (a) Cl 2p, (b) O 1s, (c) N 1s, and (d) Ni $2p_{3/2}$ in NiHC-pz-300 after the catalysis.

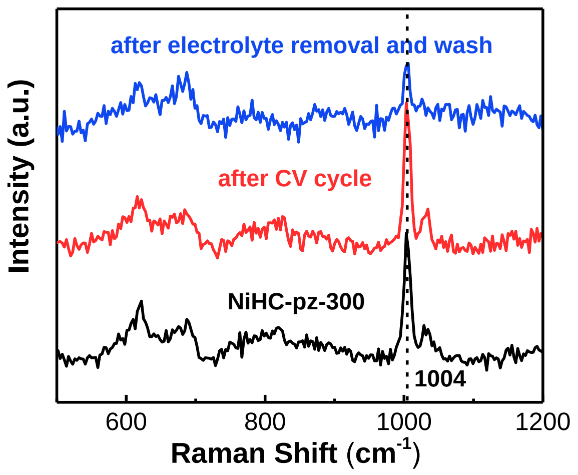

Supplementary Fig. 37. The Raman spectra of the NiHC-pz-300 material before and after CV cycles in 1 M KOH + 0.1 M benzyl alcohol, and the material removed from the electrolyte and washes with water after CV cycling.

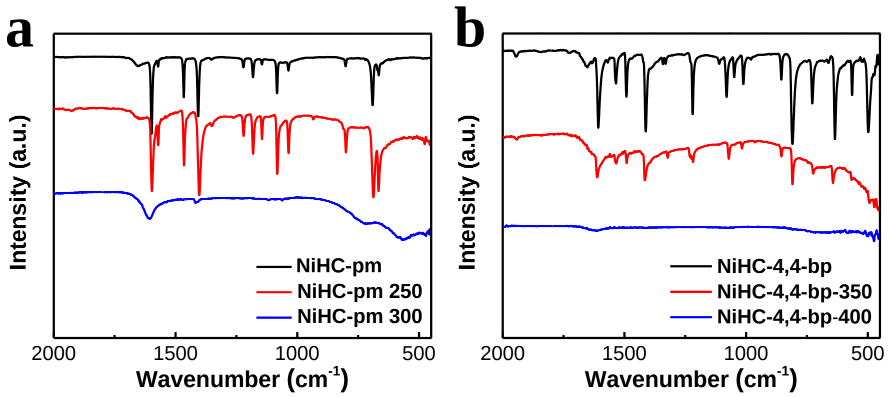

Supplementary Fig. 38. The FTIR spectra of (a) NiHC-pm, (b) NiHC-4,4-bp and their annealed products.

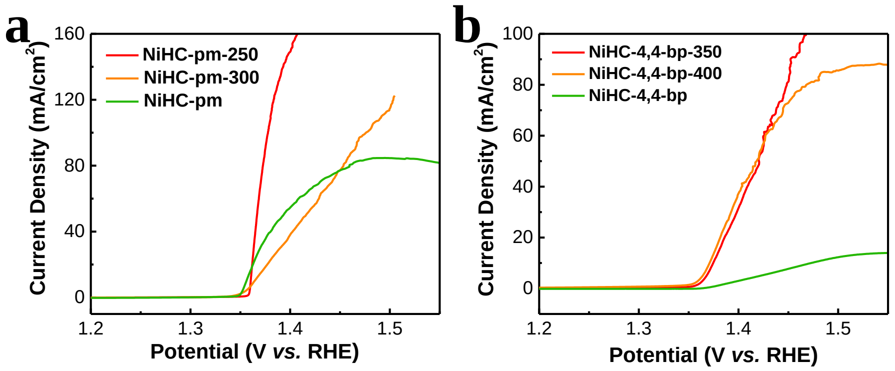

Supplementary Fig. 39. The iR compensated polarization curves of (a) NiHC-pm-250, NiHC-pm-300, NiHC-pm and (b) NiHC-4,4-bp-350, NiHC-4,4-bp-400, NiHC-4,4-bp in 1 M KOH and 0.1 M urea at a scan rate of 5 mV s^{-1}.

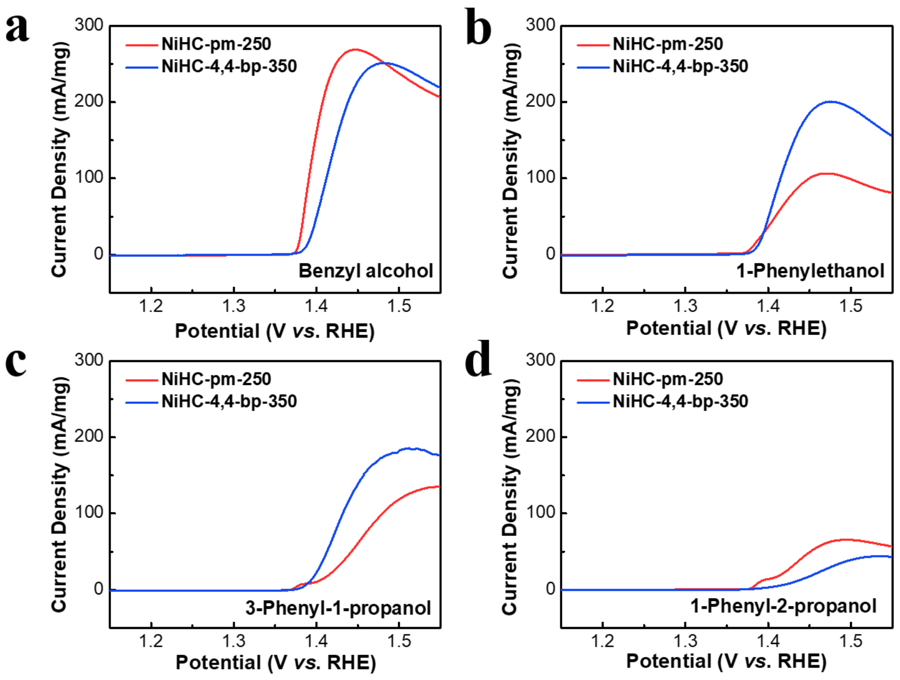

Supplementary Fig. 40. The iR compensated polarization curves of NiHC-pm-250, NiHC-4,4-BP-350 in 1 M KOH and 0.1 M (a) benzyl alcohol, (b) 1-phenylethanol, (c) 3-phenyl-1-propanol and (d) 1-phenyl-2-propanol at a scan rate of 5 mV s^{-1}.

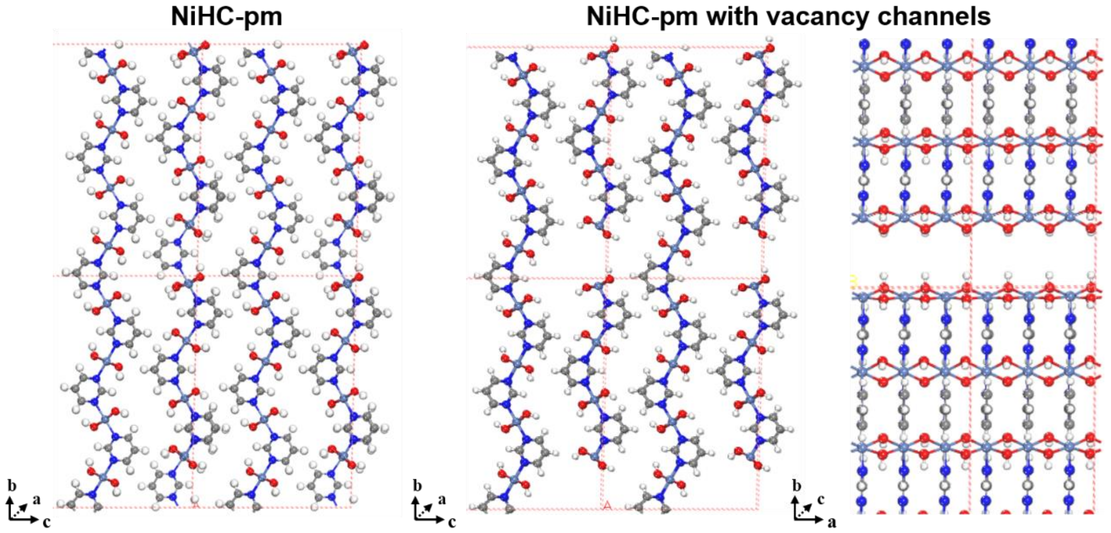

Supplementary Fig. 41. Schematic illustration of the NiHC-pm (hydroxide substitution) structure without and with vacancy channels.

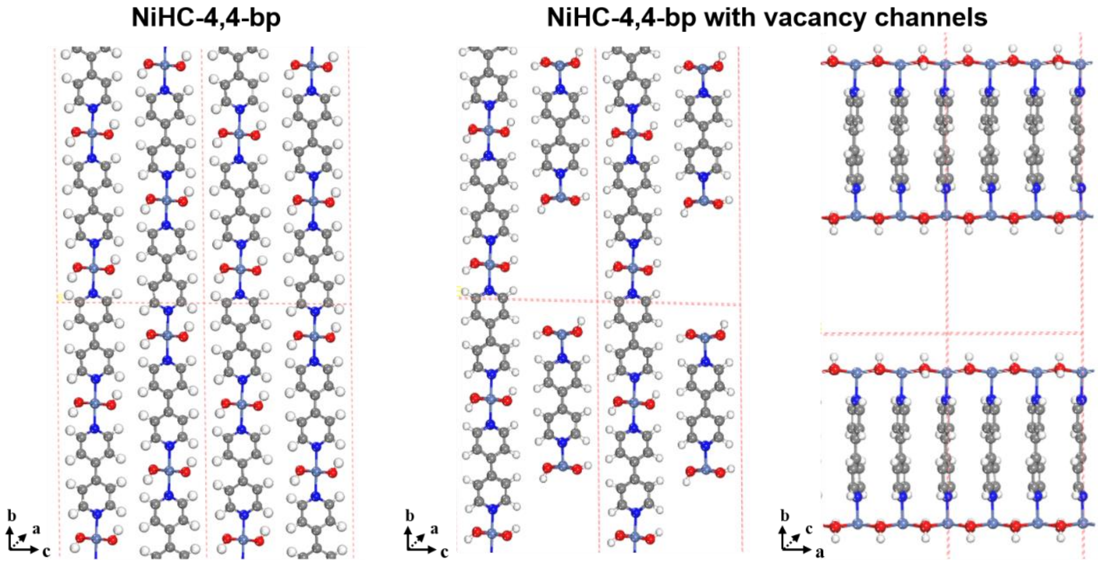

Supplementary Fig. 42. Schematic illustration of the NiHC-4,4-bp (hydroxide substitution) structure without and with vacancy channels.

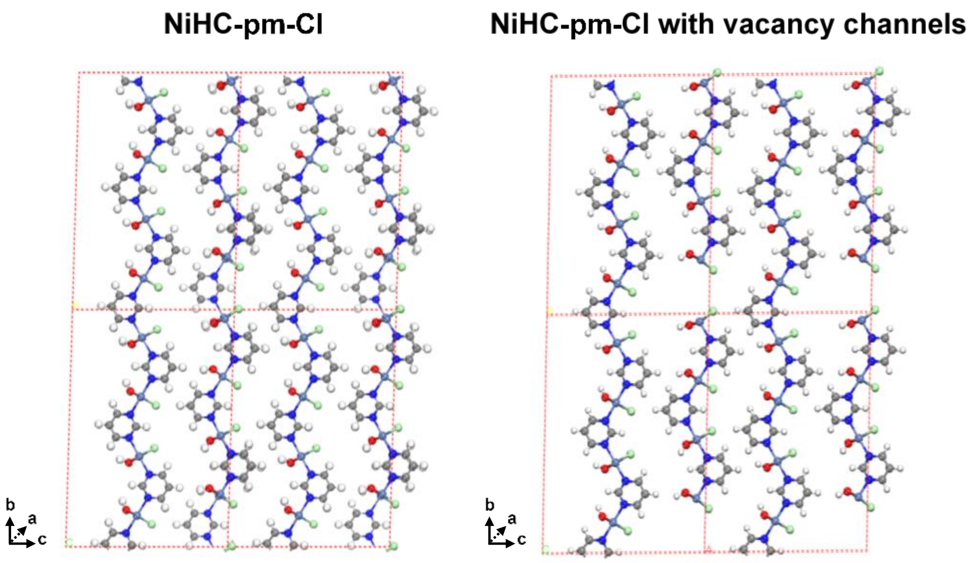

Supplementary Fig. 43. Schematic illustration of the NiHC-pm-Cl structure without and with vacancy channels.

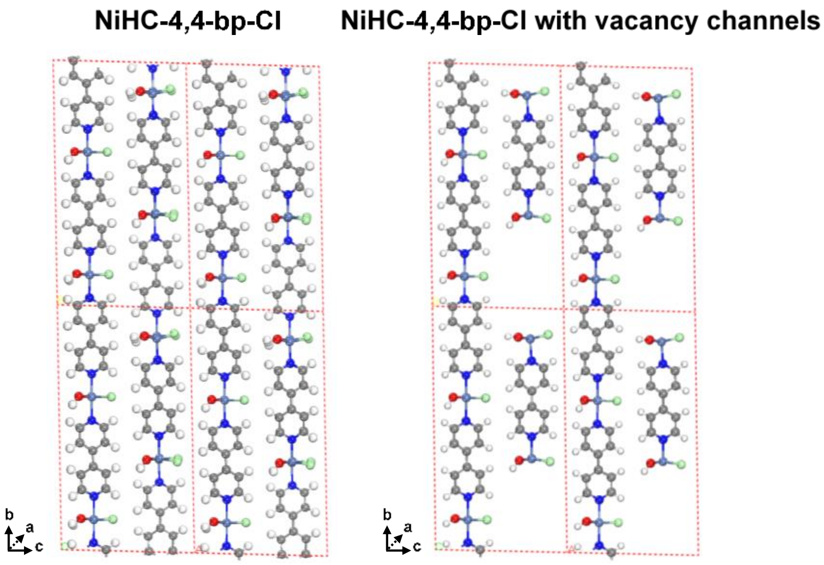

Supplementary Fig. 44. Schematic illustration of the NiHC-4,4-bp-Cl structure without and with vacancy channels.

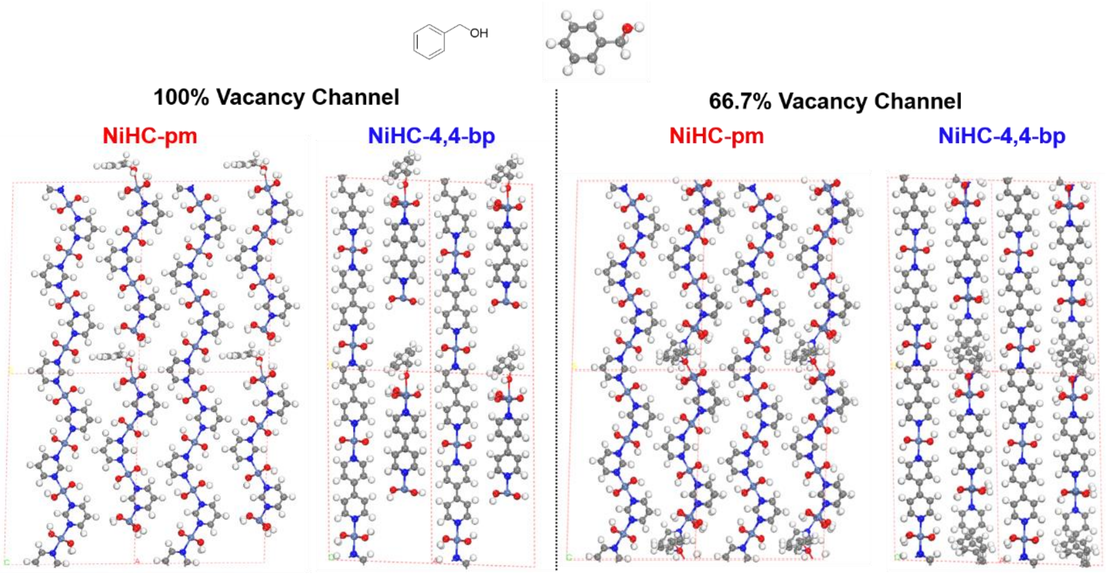

Supplementary Fig. 45. Schematic illustration of the adsorption configuration of the benzyl alcohol on NiHC-pm and NiHC-4,4-bp (hydroxide substitution) with 100% and 66.7% vacancy channels

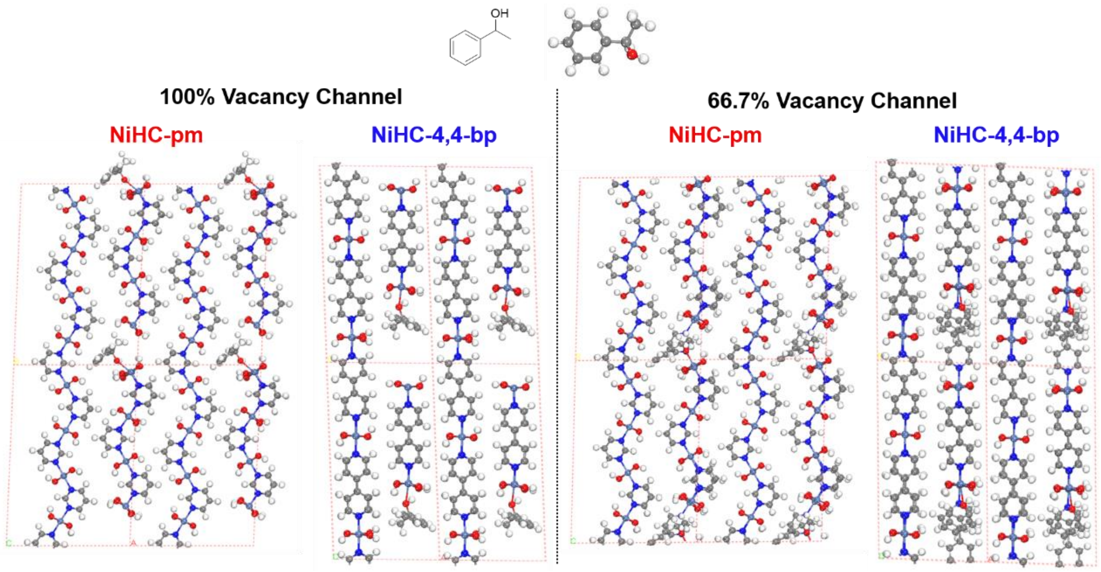

Supplementary Fig. 46. Schematic illustration of the adsorption configuration of the 1-phenylethanol on NiHC-pm and NiHC-4,4-bp (hydroxide substitution) with 100% and 66.7% vacancy channels

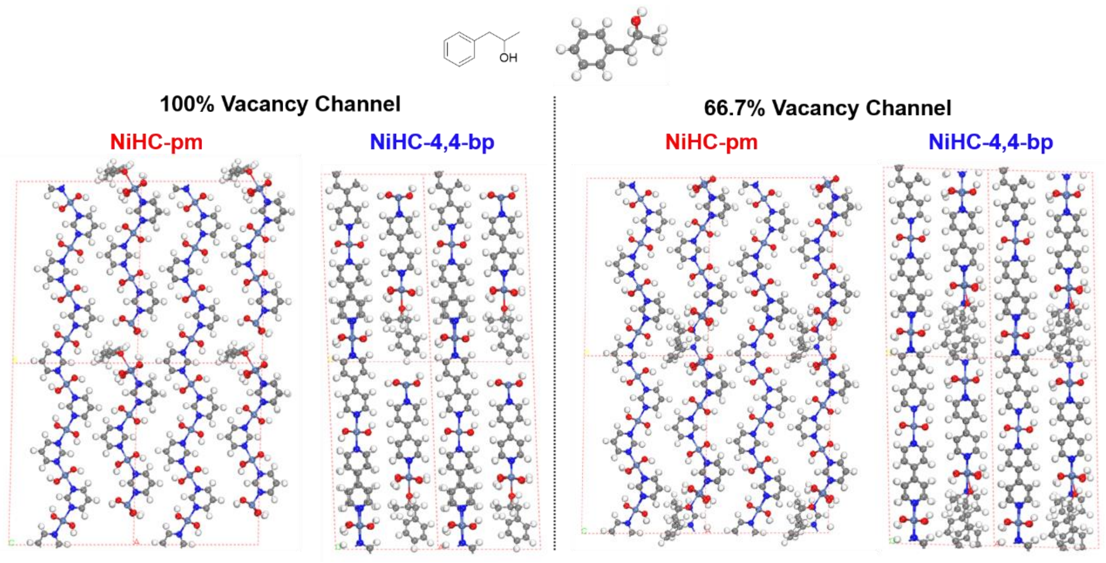

Supplementary Fig. 47. Schematic illustration of the adsorption configuration of the 1-phenyl-2-propanol on NiHC-pm and NiHC-4,4-bp (hydroxide substitution) with 100% and 66.7% vacancy channels

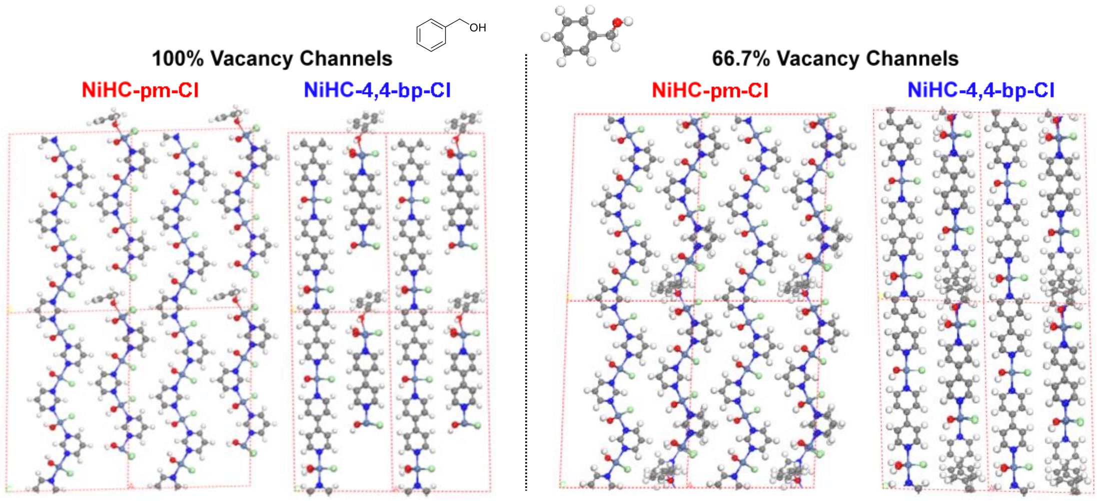

Supplementary Fig. 48. Schematic illustration of the adsorption configuration of the benzyl alcohol on NiHC-pm-Cl and NiHC-4,4-bp-Cl with 100% and 66.7% vacancy channels.

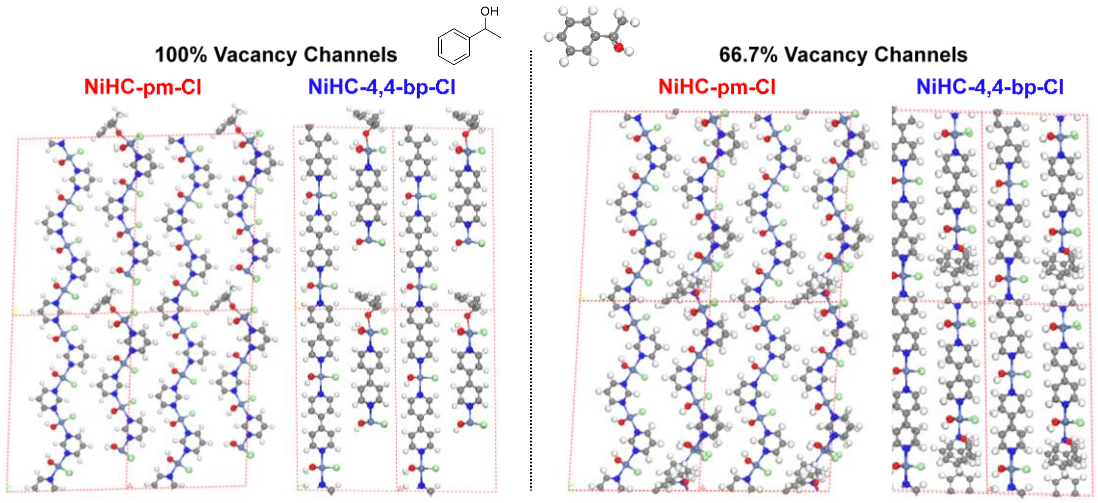

Supplementary Fig. 49. Schematic illustration of the adsorption configuration of the 1-phenylethanol on NiHC-pm-Cl and NiHC-4,4-bp-Cl with 100% and 66.7% vacancy channels.

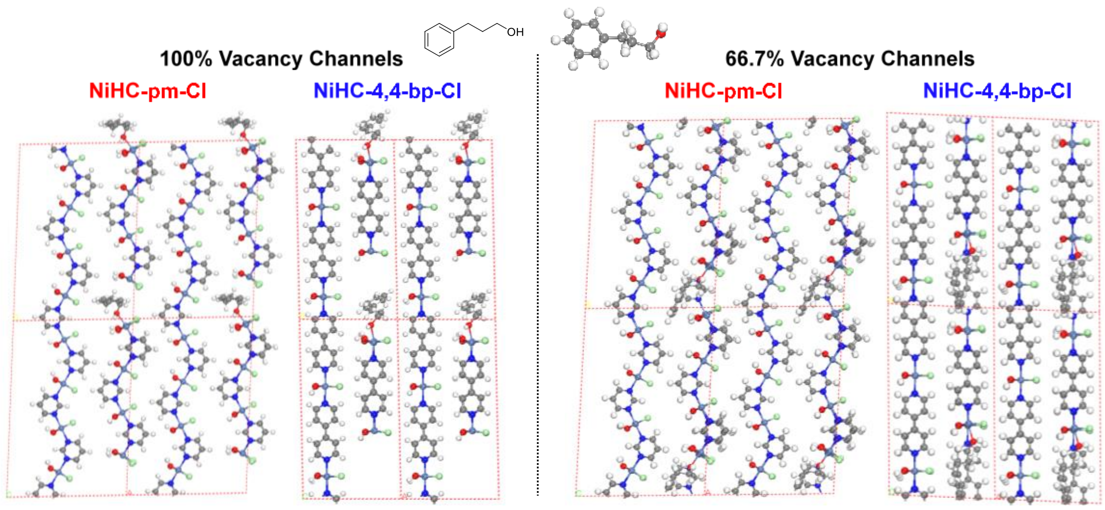

Supplementary Fig. 50. Schematic illustration of the adsorption configuration of the 3-phenyl-1-propanol on NiHC-pm-Cl and NiHC-4,4-bp-Cl with 100% and 66.7% vacancy channels.

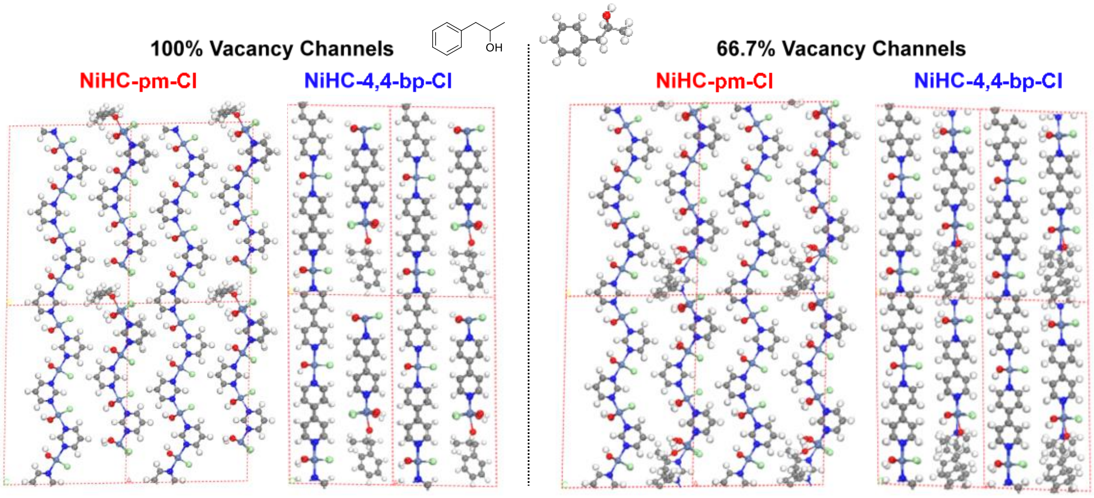

Supplementary Fig. 51. Schematic illustration of the adsorption configuration of the 1-phenyl-2-propanol on NiHC-pm-Cl and NiHC-4,4-bp-Cl with 100% and 66.7% vacancy channels.

Table S1. The liquid products after electrolysis of different substrates (1 mmol) and their calculated Faradaic efficiencies for NiHC-pz-300 (1 mg/cm^2).

**Substrates**	**Liquid products**	**Conditions (*vs.* RHE)**	**Faradaic efficiency (%)**	**Yield of liquid products (μmol)**
Methanol	Formate	1.45 V / 1 h	38.11	110.50
Ethanol	Acetate	1.45 V / 1 h	83.51	232.83
Benzyl alcohol	Benzoate	1.45 V / 1 h	87.59	464.42
1,1,1-trifluoro-2-propanol	1,1,1-Trifluoro-acetone	1.50 V / 1 h	83.61	206.37
1,4-butanediol	Succinate	1.50 V / 1 h	52.31	177.22
	4-hydroxy-butyrate		14.22	96.35
Benzylamine	Benzoate	1.45 V / 1 h	48.99	271.26
Glycerol	Formate	1.45 V / 1 h	42.88	274.80
Cyclohexanol	Adipate	1.50 V / 1 h	30.44	28.55

Table S2. The liquid products after electrolysis of different substrates (1 mmol) and their calculated Faradaic efficiencies for NiHC-pz-300 (0.1 mg/cm^2).

**Substrates**	**Liquid products**	**Conditions (*vs*. RHE)**	**Faradaic efficiency (%)**	**Yield of liquid products (μmol)**
Methanol	Formate	1.45 V / 1 h	45.28	54.20
Ethanol	Acetate	1.45 V / 1 h	76.12	127.81
Benzyl alcohol	Benzoate	1.45 V / 1 h	63.78	242.77
1,1,1-trifluoro-2-propanol	1,1,1-Trifluoro-acetone	1.50 V / 1 h	76.69	53.53
1,4-butanediol	Succinate	1.50 V / 1 h	45.35	101.99
	4-hydroxy-butyrate		32.49	146.14
Benzylamine	Benzoate	1.45 V / 1 h	41.36	146.71
Glycerol	Formate	1.45 V / 1 h	44.41	54.40
Cyclohexanol	Adipate	1.50 V / 1 h	28.17	16.17

Table S3. The liquid products after electrolysis of different substrates (1 mmol) and their calculated Faradaic efficiencies for β-$Ni(OH)_2$ (1 mg/cm^2).

**Substrates**	**Liquid products**	**Conditions (*vs*. RHE)**	**Faradaic efficiency (%)**	**Yield of liquid products (μmol)**
Methanol	Formate	1.45 V / 1 h	47.74	8.60
Ethanol	Acetate	1.45 V / 1 h	62.62	23.77
Benzyl alcohol	Benzoate	1.45 V / 1 h	31.92	23.00
1,1,1-trifluoro-2-propanol	1,1,1-Trifluoro-acetone	1.50 V / 1 h	79.64	65.17
1,4-butanediol	Succinate	1.50 V / 1 h	30.50	24.42
	4-hydroxy-butyrate		33.62	53.85
Benzylamine	Benzoate	1.45 V / 1 h	49.68	18.60
Glycerol	Formate	1.45 V / 1 h	32.96	3.40
Cyclohexanol	Adipate	1.50 V / 1 h	8.46	4.63
