# Supplementary material for: Ligand vacancy channels in pillared inorganic-organic hybrids for electrocatalytic organic oxidation with enzyme-like activities
Source: Nat Commun. 2023 Mar 2;14:1184. doi: 10.1038/s41467-023-36830-4 (PMC9981682; doi:10.1038/s41467-023-36830-4)
Supplement: Supplementary file 2 — Dataset 1 [file 41467_2023_36830_MOESM2_ESM.pdf]

Supplementary Information

Ligand Vacancy Channels in Pillared Inorganic-Organic Hybrids for Electrocatalytic Organic Oxidation with Enzyme-like Activities

Zhe Chen[1‡], Jili Li[1‡], Lingshen Meng[1], Jianan Li[2], Yaming Hao[1], Tao Jiang[1], Xuejing Yang[2], Yefei Li[1*], Zhi-Pan Liu[1], Ming Gong[1*]

[1]Department of Chemistry and Shanghai Key Laboratory of Molecular Catalysis and Innovative Materials, Fudan University, Shanghai, China, 200438

[2] National Engineering Laboratory for Industrial Wastewater Treatment, East China University of Science and Technology, Shanghai, China, 200237

[‡]These authors contributed equally to this work.

To whom correspondence may be addressed. Email: gongm@fudan.edu.cn; yefeil@fudan.edu.cn

Theoretical calculation details

Crystal Structure for bulk of NiHC-pm (hydroxide substitution) in VASP POSCAR format

```
1.0000000000
  16.16985302000     0.00000000000     0.00000000000
   0.50213885556    23.88590617338     0.00000000000
  -0.19620462552     0.10930822969     9.72684750833
C      H      N      Ni      O
84     132    42     24      48
D
   0.74757185099     0.59820015943     0.80461782364
   0.74740345399     0.59817657586     0.47145254391
   0.74765340756     0.59792933346     0.13791717870
   0.79152806031     0.64751519389     0.80366717370
   0.79134115065     0.64750265293     0.47066814855
   0.79392356360     0.54858385158     0.80096907753
   0.79150059585     0.64730236470     0.13713368199
   0.79375695044     0.54856321448     0.46729252557
   0.79407709753     0.54836240616     0.13403241964
   0.91464566925     0.59851092935     0.79756553574
   0.91445518748     0.59849774178     0.46385326984
   0.91464483177     0.59838195039     0.13079381237
   0.97184856734     0.84360668734     0.79828572200
   0.96908095782     0.34852720450     0.80116000156
   0.97167022065     0.84363366289     0.46504842870
   0.96899248303     0.34849757448     0.46795894837
   0.97184395142     0.84361399554     0.13164767134
   0.96925461303     0.34825035952     0.13447724557
   0.09420618190     0.79309559378     0.80466388214
   0.09221316212     0.29781285218     0.80237961912
   0.09356967108     0.89236743150     0.81171668552
```

0.09009832209	0.39723403377	0.80836059415
0.09411257543	0.79324903998	0.47136092203
0.09215102513	0.29782877777	0.46873899288
0.09322896235	0.89252874252	0.47820082972
0.08998003677	0.39724969408	0.47580732021
0.09423352531	0.79314689153	0.13808737218
0.09235899806	0.29739609377	0.13562960475
0.09343416565	0.89245362781	0.14511588012
0.09030286722	0.39685359429	0.14219044946
0.13846713891	0.84220672709	0.81181936976
0.13541639178	0.34721971691	0.80711055800
0.13828465843	0.84243547308	0.47828923883
0.13534149703	0.34724518369	0.47387785303
0.13844833514	0.84231950914	0.14513273780
0.13559043206	0.34677191961	0.14071178024
0.24126882648	0.00771604775	0.99789048504
0.24762748743	0.50976211400	0.99887981504
0.24139731752	0.00760306179	0.66451323550
0.24790388952	0.50968886472	0.66584820606
0.24113295079	0.00764093933	0.33122278305
0.24760936751	0.50984049751	0.33211112503
0.28462703909	0.95690833572	0.00047015274
0.28915435319	0.45829468921	0.00077377372
0.29555748988	0.55737158931	0.66923192529
0.28478569305	0.95680169550	0.66718474063
0.28832950236	0.05570424477	0.66877203219
0.28954066433	0.45824717602	0.66763397025
0.29534903174	0.55751040636	0.33566573231
0.28459790265	0.95685497064	0.33388655021
0.28804854337	0.05576087126	0.33530129481
0.28915981236	0.45839009632	0.33411484772
0.29535864576	0.55741952937	0.00248738083
0.28825899732	0.05581636246	0.00205824765
0.40827026019	0.00203898075	0.67643883965

0.41393054962	0.50218508855	0.67530730788
0.40804433983	0.00215691591	0.34293400489
0.41365496894	0.50222131965	0.34202585351
0.40818327563	0.00211306689	0.00978334799
0.41366719461	0.50217669428	0.00866761733
0.46152997265	0.75677510981	0.67978635178
0.46343559944	0.25556351403	0.67670826466
0.46140138816	0.75682239798	0.34637247362
0.46329309715	0.25558778321	0.34333050972
0.46135016004	0.75681280854	0.01314035836
0.46343121910	0.25558242933	0.00998756893
0.58013693403	0.70246576166	0.66648595585
0.58026243816	0.19900863777	0.67715723500
0.57997647165	0.70244790508	0.33323244383
0.58011004792	0.19900620562	0.34374369302
0.58561652270	0.80166623957	0.67631864860
0.58913305667	0.29817657416	0.68036774354
0.57989996119	0.70244679834	0.00001364954
0.58028523901	0.19904871667	0.01058012382
0.58552069470	0.80167116904	0.34292214380
0.58905894154	0.29815618327	0.34695305618
0.58543866556	0.80166801395	0.00970888080
0.58913535286	0.29819733070	0.01366052433
0.62714672958	0.75046896075	0.66769626361
0.62917638324	0.24621167352	0.67961775275
0.62699028720	0.75045152951	0.33448640191
0.62907564455	0.24617940864	0.34620502523
0.62689428070	0.75045503926	0.00121241533
0.62919285468	0.24623374636	0.01295861911
0.68005795990	0.59845217734	0.80844748006
0.67991609988	0.59842112420	0.47545943493
0.68011254585	0.59812128464	0.14151973595
0.69434141758	0.74851654005	0.66271375330
0.69655599296	0.24312897688	0.68142442197

0.69418564299	0.74844575432	0.32939253116
0.69644950275	0.24306959682	0.34795649835
0.69410245280	0.74845043233	0.99614386056
0.69657193423	0.24311474138	0.01474633635
0.76008018649	0.68840164663	0.80608464996
0.75988530784	0.68836339828	0.47349707817
0.75995957727	0.68814539168	0.13956604746
0.76698047918	0.50717778219	0.80063770809
0.76678705493	0.50716579839	0.46653768347
0.76715882616	0.50691830760	0.13348459908
0.82069176425	0.74142301335	0.95888502405
0.82560175299	0.22698808602	0.95728780183
0.82083773801	0.74137595211	0.62589997898
0.82548815320	0.22698431997	0.62419622512
0.82085390774	0.74131555627	0.29246476739
0.82551801118	0.22687121610	0.29080091125
0.82601986904	0.93834825639	0.96476792578
0.85124514763	0.41211769546	0.96686173778
0.82671383292	0.93746898378	0.63149074207
0.85095774372	0.41250653557	0.63374109841
0.82673078002	0.93734805882	0.29772356013
0.85137855869	0.41221031661	0.30039378488
0.90456142031	0.84355192483	0.79298758446
0.90162864580	0.34811105328	0.79926242190
0.90438290936	0.84348163123	0.45979650037
0.90155498966	0.34806951702	0.46601554811
0.90456909911	0.84351421286	0.12616530031
0.90182907191	0.34778422744	0.13252956129
0.98182356802	0.59838517823	0.79493460305
0.98161413254	0.59836871719	0.46119616239
0.98182969519	0.59829097694	0.12844778917
0.06824627143	0.69092994208	0.96924003218
0.05733548210	0.17487603306	0.96681557853
0.06794216391	0.69113969660	0.63569644797

0.05753484787	0.17497531276	0.63332641904
0.06239186098	0.48376877311	0.97240962767
0.01550414601	0.03476375248	0.96831924125
0.06820427747	0.69100805190	0.30257779733
0.05720515683	0.17495726334	0.30023595399
0.06256709536	0.48615943112	0.63893083023
0.01503899886	0.03486811830	0.63415688030
0.06245114094	0.48417520599	0.30593073732
0.01481858398	0.03545527322	0.30157445148
0.12491640226	0.75201105474	0.80363185311
0.12284551567	0.25682223124	0.80052762432
0.12096650514	0.93358447679	0.81774891795
0.11939303539	0.43794635496	0.81176901912
0.12494561243	0.75219720470	0.47058395200
0.12281456930	0.25685489885	0.46654462426
0.12050703013	0.93377116412	0.48403509626
0.11923423593	0.43796313766	0.47994418757
0.12498696937	0.75207167407	0.13726107003
0.12299522124	0.25642317783	0.13343868719
0.12085616288	0.93366968401	0.15073201680
0.11962293034	0.43752986323	0.14616397109
0.20591955533	0.84041967086	0.81717796359
0.20289729907	0.34587902574	0.80960315675
0.20572294835	0.84072225325	0.48363919286
0.20281115748	0.34592790688	0.47659506115
0.20590053336	0.84055930003	0.15036180229
0.20303607077	0.34542643511	0.14338194012
0.18023093213	0.51346860134	0.99513583560
0.17368217299	0.00935496454	0.99222958772
0.18051751140	0.51340105028	0.66224705111
0.17375681221	0.00927439098	0.65898302019
0.18023261153	0.51357850254	0.32829507580
0.17351119246	0.00927129030	0.32562985400
0.25298521870	0.91696149556	0.99753158284

0.25630056168	0.41879007894	0.99791493602
0.26944755548	0.59971721490	0.66880207019
0.25311203246	0.91685973301	0.66429615098
0.26184082021	0.09798093232	0.66728568382
0.25671443574	0.41874665156	0.66484868098
0.26930165487	0.59984973044	0.33568131876
0.25295317739	0.91691091717	0.33103213927
0.26152064779	0.09801910327	0.33409368248
0.25628756529	0.41889795701	0.33110377573
0.26925697862	0.59972518131	0.00209485951
0.26176366098	0.09808380759	0.00066605948
0.34517643919	0.69104233527	0.83731353226
0.31770137716	0.86957709061	0.84207692657
0.34451690729	0.19195653411	0.83824166052
0.31960659561	0.36706004722	0.84059164133
0.34546454319	0.69113885335	0.50431006180
0.31767696232	0.86951174543	0.50897133072
0.34423914679	0.19186543110	0.50487686003
0.31966071619	0.36711518477	0.50708379367
0.34512978028	0.69120637872	0.17085790865
0.31758948231	0.86974789196	0.17544546468
0.34453495789	0.19192113891	0.17155840374
0.31957709012	0.36716248949	0.17364416832
0.39436114443	0.76069634958	0.68363082831
0.39629561033	0.26059622415	0.67566204388
0.39424543913	0.76075874154	0.35021809616
0.39616074977	0.26066951729	0.34244579625
0.39419755202	0.76073860321	0.01698625386
0.39627611910	0.26061033972	0.00887527484
0.47555503113	0.99856912301	0.68080369990
0.48111794433	0.49784640096	0.67961046674
0.47532639214	0.99870306836	0.34747383776
0.48083744553	0.49786297895	0.34648275468
0.47545928479	0.99864730228	0.01422883107

0.48083315418	0.49781039531	0.01313259243
0.52575681022	0.56569062821	0.84481580738
0.52638783984	0.06654202372	0.84629952489
0.52584138279	0.56584497361	0.51111187964
0.52629712920	0.06636205686	0.51297994162
0.52556044113	0.56580754737	0.17831285250
0.52627224432	0.06639932050	0.17967699756
0.56163907157	0.90477833162	0.84801877720
0.56604030544	0.40235615609	0.84672528833
0.56198642577	0.90484909161	0.51443836214
0.56603610888	0.40233755488	0.51306721625
0.56151575247	0.90489991038	0.18118912417
0.56572858221	0.40239271301	0.17981873156
0.60531606326	0.66003113362	0.99280920655
0.60499350184	0.15630937153	0.00944726325
0.60560208632	0.66007128910	0.65936097430
0.60503859210	0.15630213989	0.67608209539
0.60533904313	0.66002700270	0.32597046682
0.60483462054	0.15626994041	0.34263281676
0.61814582407	0.84123536012	0.67897708711
0.62315262783	0.33718738245	0.68227006389
0.61811736008	0.84120988222	0.34557926904
0.62312262934	0.33712673469	0.34887924531
0.61801924624	0.84122622412	0.01246303295
0.62322340106	0.33716722680	0.01555681590
0.87453231584	0.64827748410	0.79950417063
0.87433884048	0.64825846394	0.46606658346
0.87697669879	0.54914182283	0.79838589649
0.87449957443	0.64814194278	0.13292568840
0.87679575576	0.54911699976	0.46433791618
0.87711241142	0.54897911433	0.13132079720
0.01101539080	0.79366103221	0.79851349200
0.01010550792	0.89287730788	0.80443216307
0.00819669128	0.29845487554	0.79926099883

0.00603341345	0.39806358213	0.80542923905
0.01094034037	0.79373191688	0.46531868358
0.00973682510	0.89295940642	0.47111768053
0.00814636065	0.29843322952	0.46577194359
0.00592638830	0.39803513095	0.47270093492
0.01105302529	0.79369392123	0.13184389792
0.00996277523	0.89293424686	0.13807625049
0.00834539788	0.29814971264	0.13247978010
0.00623462444	0.39775877888	0.13910344249
0.37890360030	0.55352480590	0.67366066358
0.36790537635	0.95359565260	0.67345876846
0.37176854281	0.05287176741	0.67456622985
0.37264989133	0.45406806835	0.67261787351
0.37872425895	0.55358322528	0.34030661755
0.36773170235	0.95367722877	0.34018499757
0.37147964344	0.05294967474	0.34088152777
0.37227400704	0.45413350339	0.33932194261
0.37875807239	0.55353834271	0.00720466985
0.36776581503	0.95366800386	0.00688995946
0.37169098102	0.05294198632	0.00767000983
0.37226736327	0.45407739203	0.00575749542
0.49694622185	0.70571009583	0.67295755972
0.49696022506	0.20389344526	0.67563225890
0.49677923449	0.70573656315	0.33968525991
0.49681373493	0.20392095879	0.34224977025
0.50265235109	0.80503571667	0.68164011611
0.50615032977	0.30317428533	0.67894978906
0.49670074521	0.70571870579	0.00643639359
0.49697288100	0.20393452211	0.00903270879
0.50254233845	0.80507089096	0.34806830755
0.50605507520	0.30319496230	0.34554188077
0.50248175132	0.80506928323	0.01493491060
0.50613265119	0.30320918697	0.01228153735
0.94378711993	0.72164072836	0.79792236018

0.94823516117	0.22683425501	0.79600778653
0.94377731321	0.72171826268	0.46466676044
0.95049646108	0.96604829586	0.80161074144
0.94814827832	0.22684666152	0.46287278053
0.94015686186	0.47390670191	0.80213607382
0.94382384023	0.72166296427	0.13137551256
0.95002357489	0.96617045443	0.46832197504
0.94811441676	0.22674605517	0.12964196847
0.94015751489	0.47401660629	0.46855939986
0.95027304046	0.96619221194	0.13531466211
0.94029973147	0.47371425848	0.13532553676
0.43855227913	0.62956265000	0.67325704648
0.43325656440	0.12840799773	0.67509469768
0.43836010604	0.62969949487	0.33999449320
0.43300658576	0.12839113466	0.34161534013
0.43769320254	0.87938636286	0.67864866796
0.43838784428	0.62964162821	0.00672775346
0.44160524896	0.37866791911	0.67648687042
0.43326695025	0.12850486936	0.00841887877
0.43756557072	0.87944119650	0.34529618184
0.44147348974	0.37880129401	0.34310304085
0.43749647173	0.87946378994	0.01199048498
0.44142908160	0.37878965547	0.00977246728
0.87622995580	0.75621225893	0.96111115549
0.87305276156	0.25136555900	0.96033478740
0.87695440409	0.95927969703	0.96598944169
0.86531932590	0.45152974570	0.96745182989
0.87628859016	0.75633548830	0.62750452608
0.87300465155	0.25132697780	0.62697723376
0.87654961408	0.95951695496	0.63324144218
0.86544709332	0.45181852266	0.63378423973
0.87616104649	0.75643709798	0.29446393588
0.87292638355	0.25133533825	0.29373372427
0.87621543697	0.95978969373	0.29934536925

0.86553491040	0.45159915232	0.30029828385
0.00835404399	0.68776494045	0.96799872579
0.00010912599	0.18834784791	0.96552529563
0.00807910054	0.68768058908	0.63445829532
0.00920256466	0.50358620064	0.97083612557
0.00020097031	0.18830496373	0.63224537115
0.02303283556	0.99417682270	0.97067576344
0.00835524727	0.68778846384	0.30143570859
0.00822944814	0.50444315661	0.63742203738
0.99996455720	0.18826480595	0.29924794662
0.02270742915	0.99431595028	0.63691194210
0.00918648244	0.50383876244	0.30422800773
0.02244498133	0.99486789709	0.30403986240
0.36117026338	0.65156114512	0.83638873119
0.35734953455	0.15194365153	0.83905446292
0.36125135679	0.65161100775	0.50323084934
0.35713048851	0.15185756054	0.50568502907
0.36115760621	0.65173580742	0.17006998604
0.36861755857	0.84723281426	0.84343774239
0.35713217142	0.15187038592	0.17236646823
0.37229088296	0.34674277748	0.84196323660
0.36861389321	0.84720756173	0.51035893014
0.37239145686	0.34685137758	0.50882697552
0.36842799962	0.84730060826	0.17689440783
0.37235342665	0.34692382255	0.17549856215
0.51737520691	0.60616119206	0.84317990304
0.50252634779	0.91325639558	0.84666753056
0.50901450700	0.10582908278	0.84511597657
0.50722626962	0.41187812312	0.84414594975
0.51696645194	0.60627578425	0.50988790579
0.50282820021	0.91322384347	0.51331510715
0.50861965840	0.10560313551	0.51126254449
0.50724906656	0.41189371918	0.51062250639
0.51718467895	0.60625764393	0.17645513323

0.50237575320	0.91326598995	0.17998572163
0.50855291949	0.10561297970	0.17811915315
0.50706555742	0.41221116321	0.17763759445

Crystal Structure for bulk of NiHC-4,4-bp (hydroxide substitution) in VASP POSCAR format

1.0000000000

12.07653208000	0.00000000000	0.00000000000
-0.70982776031	22.63094696551	0.00000000000
0.02658093692	-0.02233421070	9.94780290579

C	H	N	Ni	O
90	96	18	12	24

D

0.15200063956	0.99949391311	0.01830187427
0.15324054121	0.49943032203	0.02181119842
0.65824731465	0.75392962063	0.01404537166
0.15243626166	0.99944162315	0.35108460703
0.15359906400	0.49930448258	0.35407836488
0.65755709991	0.75403225644	0.34897247089
0.15213380959	0.99976502219	0.68540479486
0.15261134822	0.49967619200	0.68847402787
0.65765955658	0.75407870274	0.68174312200
0.16189891293	0.24574398587	0.31051687033
0.16181986198	0.74614479121	0.30645419475
0.65435039550	0.50185395748	0.30072991025
0.16212599278	0.24613252420	0.64293360469
0.16277045519	0.74675911596	0.63867846410
0.65317184271	0.50204300136	0.63393653340
0.16189123318	0.24613565937	0.97549194309
0.16293593184	0.74659776910	0.97207500818
0.65439396602	0.50179875021	0.96747441502
0.15566375512	0.30691247454	0.30817683292
0.15612900721	0.80735333026	0.30354923460
0.65177226738	0.56299254106	0.29703417227

0.15557149477	0.30723969834	0.64072811649
0.15710937363	0.80798493423	0.63587403057
0.65106647944	0.56318851291	0.63012619313
0.15593203259	0.30732789302	0.97300268920
0.15728687723	0.80782890366	0.96909513883
0.65191226637	0.56293415993	0.96379047613
0.15134402097	0.43814428262	0.02020457451
0.15048327600	0.93815976870	0.01763118235
0.65349477345	0.69284626460	0.01077917486
0.15142165916	0.43803335364	0.35274384487
0.15023279914	0.93808759521	0.35049285435
0.65310886083	0.69292283719	0.34532259239
0.15038571084	0.43838230085	0.68735893370
0.15040484807	0.93841773050	0.68551365468
0.65305090274	0.69298476085	0.67776049524
0.25140000102	0.34261546256	0.32922196218
0.25107481793	0.84306707324	0.32902151681
0.74923032563	0.59717111123	0.32092016400
0.25090595149	0.34309201014	0.66275575486
0.25186938428	0.84364644470	0.66236491660
0.74880642024	0.59722817350	0.65336169540
0.25149727048	0.34295900969	0.99523676874
0.25204470619	0.84348658698	0.99547055282
0.74946928558	0.59706457625	0.98777509979
0.24931336799	0.40772700416	0.32847023981
0.24913054667	0.90820890850	0.32867778634
0.75033015130	0.66189138943	0.32087953704
0.24828802697	0.40819446970	0.66235913512
0.24933762388	0.90876960765	0.66255417286
0.75021528098	0.66192074009	0.65329487280
0.24930659531	0.40808063917	0.99500161430
0.24961088211	0.90858321867	0.99528936528
0.75072813441	0.66173818116	0.98731574399
0.33979786999	0.00476239917	0.30959885084

0.33961637550	0.50423870712	0.30609735766
0.84458918304	0.75740974449	0.30123167501
0.33927114962	0.00542904847	0.64255854308
0.33859104949	0.50473457525	0.64032494163
0.84477841742	0.75735298750	0.63436610473
0.33932474960	0.00532409804	0.97679102039
0.33915373156	0.50472219111	0.97362362692
0.84554519269	0.75707512140	0.96773011600
0.34850512447	0.25198641601	0.02024359197
0.34779884552	0.75219868875	0.02433771188
0.84083920431	0.50490031386	0.01773677159
0.34898116241	0.25183958202	0.35249240392
0.34686624944	0.75175733003	0.35720768834
0.84070845577	0.50499044747	0.35125820989
0.34884067902	0.25234021934	0.68680636586
0.34771337340	0.75232863801	0.69041586912
0.83961226826	0.50491369498	0.68399029479
0.35008399727	0.31333553474	0.01915591330
0.34950744171	0.81350180186	0.02263219900
0.84613294517	0.56593871698	0.01357373968
0.35030989819	0.31315053045	0.35197311639
0.34863523600	0.81305647943	0.35554566978
0.84586841779	0.56605062190	0.34713122724
0.34982702770	0.31365085348	0.68628571954
0.34944843820	0.81365898032	0.68915431577
0.84520351793	0.56596586089	0.67965885171
0.34490571801	0.44299812813	0.30353982760
0.34528572328	0.94357858413	0.30764092594
0.84776558618	0.69623561306	0.29802471149
0.34383649525	0.44355469394	0.63753651254
0.34520022680	0.94425594833	0.64045202718
0.84780011617	0.69619990136	0.63096682418
0.34484614933	0.44352637209	0.97074536402
0.34546026627	0.94417156433	0.97421095188

0.84845723787	0.69589714608	0.96502207642
0.08383493945	0.10303657093	0.17151222910
0.09580217242	0.59203719699	0.17608698288
0.08180480402	0.10393003263	0.50172735822
0.09541536067	0.59204350832	0.51011702574
0.08324666841	0.10327727162	0.83613537962
0.09563501670	0.59243175446	0.84259690742
0.08961297468	0.21687772738	0.29252407427
0.09062135209	0.71708688020	0.28444311370
0.58141856035	0.47396665376	0.27973375592
0.09011720572	0.21714645786	0.62415546536
0.09194512003	0.71756447004	0.61587588385
0.58003236320	0.47426667423	0.61322498292
0.08970852225	0.21733611007	0.95620359632
0.09216698843	0.71748034311	0.94860454478
0.58147290930	0.47390889260	0.94625524522
0.07372575927	0.41384395391	0.04085266182
0.07245065424	0.91397588159	0.03680573384
0.57504221562	0.66912264553	0.03023036884
0.07357925925	0.41387346965	0.37282396486
0.07182701254	0.91417532952	0.36906970351
0.57484573083	0.66906639959	0.36503011815
0.07261910506	0.41424654846	0.70810592004
0.07230652672	0.91434954564	0.70518092220
0.57468793243	0.66916647844	0.69702985661
0.07527753259	0.32562483290	0.28796620793
0.07750720490	0.82723414217	0.27948883119
0.57370096421	0.58354177567	0.27314044747
0.07510710940	0.32578389522	0.61994547725
0.07867083552	0.82783041008	0.61086680552
0.57307214095	0.58382328756	0.60594776224
0.07583074139	0.32610934187	0.95133492939
0.07897249454	0.82769555195	0.94361260651
0.57405214947	0.58353916907	0.93923392657

0.07576895730	0.02316691742	0.03577441777
0.07781060935	0.52324779564	0.04202131566
0.58476715619	0.77914746420	0.03472621968
0.07654142877	0.02338210931	0.36851787291
0.07824038378	0.52332380917	0.37336824414
0.58391043704	0.77907896622	0.37003834614
0.07604404139	0.02355076109	0.70271362925
0.07722674974	0.52366639096	0.70797131132
0.58399112322	0.77915469763	0.70263441216
0.92560091074	0.38897760720	0.17211368281
0.88466904718	0.92692312900	0.16532446376
0.92494528322	0.38856930151	0.50381847152
0.88545203279	0.92651394840	0.50053557837
0.92435371984	0.38935506520	0.83691071917
0.88570876630	0.92575255458	0.83294248383
0.42368057072	0.42300559262	0.27982205642
0.42562686064	0.92461552347	0.28745892325
0.92600452467	0.67548286760	0.27539848265
0.42255642104	0.42349448013	0.61400924725
0.42552374947	0.92542342998	0.61970179721
0.92607658258	0.67547605859	0.60822155367
0.42373445992	0.42360004821	0.94724527338
0.42597220197	0.92542277738	0.95384219097
0.92670363490	0.67506227330	0.94292316070
0.42439535694	0.22837223821	0.03915895195
0.42305953979	0.72857773811	0.04581144202
0.91343802414	0.47949775013	0.04093653542
0.42515071355	0.22829265078	0.37027170993
0.42225673989	0.72816228702	0.37819818423
0.91357138551	0.47977289731	0.37419533713
0.42503018167	0.22888154361	0.70515327147
0.42309971313	0.72875947902	0.71164038422
0.91216649806	0.47947263851	0.70706687734
0.41261188068	0.03328478639	0.29157733416

0.41124945352	0.53311099071	0.28471314649
0.91744220660	0.78544345209	0.28053897654
0.41182122033	0.03409405096	0.62402075511
0.41018858172	0.53364608943	0.61894791476
0.91776493140	0.78539939594	0.61443613920
0.41192416794	0.03406965379	0.95942289060
0.41065829396	0.53373043994	0.95252308009
0.91866558497	0.78501619751	0.94756959333
0.42774633441	0.33769058636	0.03913586836
0.42669011950	0.83791528483	0.04455480875
0.92476325662	0.58932850549	0.03373023121
0.42803462564	0.33755818561	0.37121362395
0.42590590516	0.83744050034	0.37688186452
0.92430241813	0.58953431192	0.36784798359
0.42734190613	0.33814112503	0.70636498064
0.42654971520	0.83808748908	0.71129596033
0.92377451940	0.58932557101	0.70019420348
0.41997499466	0.14674366908	0.17062360072
0.40470735330	0.65936228915	0.17579891113
0.41861886148	0.14799505140	0.50174544231
0.40428267003	0.65874310023	0.50838367606
0.41910982100	0.14740436749	0.83545191553
0.40393943643	0.65943118102	0.84158398586
0.61288526324	0.33523748619	0.16617322726
0.57476752754	0.86978480574	0.17072083656
0.61319236846	0.33473985825	0.50027235533
0.57515631343	0.86906323260	0.50460263563
0.61215664358	0.33591625365	0.83378689401
0.57609836932	0.86915331622	0.83775453735
0.24482738445	0.03291119504	0.33129191644
0.24576604788	0.53285777042	0.33219492629
0.75140518047	0.78659632942	0.32793553309
0.24422067351	0.03336925895	0.66459418543
0.24465388045	0.53327707733	0.66636969045

0.75153509188	0.78655548566	0.66081370422
0.24411242049	0.03319857024	0.99853179100
0.24512779026	0.53315622494	0.99966304200
0.75236051759	0.78633974871	0.99357363949
0.25711559478	0.21804055047	0.33211677164
0.25555256092	0.71781581280	0.33367686407
0.74701865575	0.47261602305	0.32878948839
0.25720792820	0.21844056769	0.66563330262
0.25644933668	0.71843042690	0.66649387164
0.74576926151	0.47269159549	0.66166898855
0.25668997543	0.21826320750	0.99890883817
0.25650479406	0.71828330769	0.00032916374
0.74706577845	0.47260286819	0.99546314918
0.25080824781	0.12574090513	0.00050274760
0.25067035949	0.62554269151	0.00370606880
0.25094119213	0.12562989921	0.33355972735
0.25070118985	0.62512382806	0.33677759549
0.25055046081	0.12584469365	0.66696164498
0.25031386365	0.62554850058	0.67028896462
0.75057796866	0.38517078533	0.33354500651
0.74939400858	0.87440834036	0.33309795222
0.74979108115	0.38532037994	0.66632720630
0.74964544279	0.87417127293	0.66610249105
0.75071022255	0.38529990941	0.99990332802
0.75016437381	0.87391490002	0.99955512068
0.84974884943	0.37321756552	0.16818664311
0.85855314460	0.88575818547	0.16549814925
0.84905615302	0.37290197883	0.50056616794
0.85778671969	0.88564122954	0.49922199254
0.84885537407	0.37312337219	0.83341721207
0.85944837321	0.88466892266	0.83240168833
0.14603246312	0.13139172863	0.16850500285
0.13860783945	0.62904083383	0.17084125390
0.14524631821	0.13148798356	0.50027572491

0.13809299336	0.62910891330	0.50481382960
0.14608677152	0.13126365538	0.83409011533
0.13843311527	0.62946365864	0.83754687180
0.35593481558	0.11961874033	0.16771819836
0.36388047658	0.62171805506	0.17027593259
0.35492978336	0.12063082802	0.50029792690
0.36258681304	0.62136455397	0.50388555588
0.35543265987	0.12001784251	0.83376726555
0.36219105753	0.62207200396	0.83723470930
0.64200412863	0.37582192774	0.16581602828
0.65065649056	0.88543670892	0.16719533396
0.64078348603	0.37561776843	0.49988256664
0.65007451987	0.88609063984	0.50111938518
0.64122472190	0.37650226512	0.83351686262
0.65131565471	0.88584144180	0.83401019035

Crystal Structure for bulk of NiHC-pm-Cl in VASP POSCAR format

1.0000000000

16.51511213000	0.00000000000	0.00000000000
0.67530986044	24.30268393757	0.00000000000
-0.17694418011	0.10849658652	10.20520122297

C	H	N	Ni	O	Cl	O	Cl
84	108	42	24	12	12	12	12

D

0.74698137105	0.59749092352	0.80459848411
0.74681858331	0.59750044126	0.47169017149
0.74684844640	0.59727388209	0.13872477368
0.78997458998	0.64585356725	0.80334398498
0.78987736722	0.64583256928	0.47034075234
0.79251005854	0.54844461305	0.80209533327
0.78977144520	0.64569482234	0.13732525684
0.79232155497	0.54844274885	0.46885057477
0.79244362827	0.54829421586	0.13556921689
0.91022003929	0.59693361023	0.79753658308

0.91008702121	0.59686529051	0.46407302762
0.91009935485	0.59685080715	0.13072554905
0.97072773210	0.84313910362	0.79925615368
0.96849712921	0.34734999058	0.80075700140
0.97060649174	0.84310736397	0.46555850847
0.96860383268	0.34732833864	0.46699718544
0.97065606629	0.84320189261	0.13162654951
0.96872878551	0.34721283743	0.13427453757
0.08990913888	0.79209951087	0.80490109450
0.08842289071	0.29699661253	0.80197071603
0.08975749679	0.88982303410	0.81119672823
0.08655886716	0.39481716721	0.80770496082
0.08969869179	0.79205664611	0.47157932459
0.08847258923	0.29688032215	0.46819689966
0.08970047312	0.88974195743	0.47795634064
0.08674078948	0.39469726758	0.47518043369
0.08982874696	0.79217085055	0.13776711407
0.08851446450	0.29662003824	0.13522010736
0.08973655019	0.88986288802	0.14423683961
0.08704263522	0.39441366693	0.14178425730
0.13360793915	0.84018492755	0.81113394936
0.13120060983	0.34541792829	0.80653810603
0.13351177779	0.84010239297	0.47791297569
0.13136004900	0.34526694948	0.47348344589
0.13359301084	0.84025103405	0.14415881508
0.13152848303	0.34496058019	0.14032711200
0.24193563558	0.00914773462	0.99819622303
0.24697157461	0.51056618861	0.99848587288
0.24189674380	0.00920470711	0.66440493331
0.24718840023	0.51049902736	0.66539551035
0.24172431385	0.00884464382	0.33165278999
0.24670697043	0.51053117357	0.33247648183
0.28574103127	0.95955148300	0.00130734845
0.28990160100	0.46060379653	0.00050471166

0.29279446451	0.55780798303	0.66828453217
0.28573077406	0.95962725086	0.66743522368
0.28679912193	0.05687235248	0.66818303804
0.29017248104	0.46055220898	0.66722734469
0.29242518168	0.55779929169	0.33567458320
0.28567745197	0.95932681339	0.33487746075
0.28649010376	0.05659240577	0.33468717798
0.28965516329	0.46057648926	0.33408593319
0.29268306478	0.55783865336	0.00156188552
0.28676902544	0.05684454668	0.00173573667
0.40551461185	0.00487970401	0.67657931031
0.41059942786	0.50482984972	0.67455556305
0.40532214866	0.00477824383	0.34298968756
0.41017100188	0.50476219955	0.34165693952
0.40548812589	0.00485858178	0.01034544706
0.41042864253	0.50477280112	0.00799979209
0.46156814600	0.75664309351	0.67916558611
0.46343550175	0.25479924131	0.67637960959
0.46155047193	0.75659237161	0.34573515415
0.46361685418	0.25484069784	0.34354715840
0.46172151133	0.75653517616	0.01221869861
0.46352169279	0.25489899728	0.01016934470
0.57939041387	0.70507140536	0.66736466535
0.57938385434	0.20064628766	0.67763804524
0.57932978621	0.70504215309	0.33418138707
0.57956128749	0.20075567126	0.34478868688
0.58101199807	0.80281585895	0.67570455607
0.58485745756	0.29838353949	0.68064520662
0.57961704890	0.70507353520	0.00060917378
0.57939593489	0.20073252233	0.01115820252
0.58098033342	0.80280336650	0.34234138746
0.58498426090	0.29851132865	0.34705038486
0.58104981444	0.80279362000	0.00914026132
0.58490590569	0.29848574159	0.01418292759

0.62373169969	0.75285644245	0.66872068555
0.62564875882	0.24758802329	0.68028495385
0.62366190066	0.75285297201	0.33542830280
0.62577081196	0.24772865436	0.34687455168
0.62388331110	0.75287145421	0.00222999464
0.62564076728	0.24767193341	0.01405201033
0.68083257850	0.59730874631	0.80863243818
0.68067536880	0.59737236291	0.47574928303
0.68070409103	0.59705910639	0.14290837046
0.68964250896	0.75163737643	0.66411138173
0.69162594070	0.24484282320	0.68236368317
0.68957925374	0.75162165481	0.33093154992
0.69175864110	0.24501922396	0.34864834551
0.68981212345	0.75176926015	0.99800231602
0.69162878081	0.24495215835	0.01616773275
0.75901588776	0.68617986677	0.80590027196
0.75902076554	0.68618103199	0.47308509245
0.75873604694	0.68597271395	0.13994621946
0.76457203050	0.50831268539	0.80302354534
0.76430627882	0.50832701764	0.46954107031
0.76455252347	0.50814109441	0.13604931149
0.82335283255	0.74734771668	0.95965761214
0.82147097039	0.23420369281	0.95790204871
0.82335268084	0.74715181687	0.62659045219
0.82112093915	0.23394902958	0.62450739420
0.82284400828	0.74718821614	0.29321831413
0.82154055004	0.23394108794	0.29094470781
0.82270864856	0.94351406617	0.96523475665
0.84298317389	0.41774619819	0.96708358036
0.82273236514	0.94277488281	0.63226411151
0.84281699810	0.41811021919	0.63362269001
0.82289037915	0.94261135902	0.29856533648
0.84289347480	0.41771729989	0.30022791007
0.90479816598	0.84409614157	0.79411280287
0.90244956900	0.34753005320	0.79886523723

0.90467046091	0.84408324382	0.46016225054
0.90257141174	0.34752912996	0.46510409372
0.90474740907	0.84415380700	0.12624309407
0.90270672063	0.34748249550	0.13223932077
0.97611059476	0.59674531448	0.79473122399
0.97598196683	0.59660196182	0.46094006343
0.97597937549	0.59669754263	0.12785660424
0.11850721229	0.75111896972	0.80437773481
0.11764654360	0.25619295418	0.80072534323
0.11773723788	0.92992826858	0.81646730995
0.11383926626	0.43514971376	0.81158858351
0.11822597262	0.75104991380	0.47107504683
0.11774113461	0.25609704808	0.46654643941
0.11766620894	0.92983597023	0.48345324362
0.11398432767	0.43503609019	0.47979866737
0.11841723207	0.75116322747	0.13778088156
0.11762950611	0.25579491792	0.13341547905
0.11778022079	0.92993873863	0.14975961273
0.11447844279	0.43469147919	0.14570247137
0.19966263076	0.83844213505	0.81584350118
0.19725854555	0.34413819285	0.80936108907
0.19957931448	0.83826464236	0.48285479133
0.19741975384	0.34407183322	0.47627778971
0.19964169134	0.83839317192	0.14916369239
0.19760238138	0.34361728878	0.14232672078
0.18081381921	0.51293085971	0.99414257165
0.17573377725	0.01088794787	0.99265395906
0.18101777447	0.51286828298	0.66124037190
0.17568841818	0.01090669138	0.65916043888
0.18054280293	0.51292013827	0.32814403193
0.17551385000	0.01051608318	0.32638227892
0.25532755243	0.92011357346	0.99869235572
0.25864225922	0.42148589222	0.99788868769
0.26516544038	0.59909031038	0.66700655913
0.25542329764	0.92016028238	0.66504091793

0.25853437219	0.09794755959	0.66643557274
0.25898791625	0.42142008542	0.66476720084
0.26472090518	0.59905362443	0.33469565150
0.25544692173	0.91981326756	0.33261195638
0.25816050714	0.09765323489	0.33259574445
0.25833927083	0.42148805688	0.33130393572
0.26501406653	0.59909759879	0.00032704980
0.25857672048	0.09795267800	0.99956666048
0.34237519552	0.68787153658	0.83646749403
0.31739752514	0.86790154920	0.84257329877
0.34082190463	0.18869320405	0.83793941973
0.32096617042	0.36694658997	0.84059876837
0.34273172752	0.68790855225	0.50327870561
0.31785148251	0.86775604091	0.50942283698
0.34058733669	0.18886280729	0.50386701307
0.32165386711	0.36673146694	0.50702593344
0.34234231438	0.68765589283	0.16983254706
0.31796675331	0.86752367152	0.17602201463
0.34072227080	0.18915868389	0.17155409087
0.32118058254	0.36694791787	0.17375455851
0.39564092914	0.75925879665	0.68301636633
0.39748891498	0.25863374090	0.67478258768
0.39563341497	0.75919224694	0.34981463560
0.39766331125	0.25864739778	0.34211046326
0.39580367553	0.75911241887	0.01619110926
0.39757287125	0.25874888176	0.00880176334
0.47147928467	0.00331680351	0.68131398491
0.47653632952	0.50260042158	0.67829191397
0.47128548363	0.00328321955	0.34774911484
0.47612164384	0.50248577455	0.34550592056
0.47146036339	0.00324206370	0.01496700140
0.47636757819	0.50248987270	0.01186039965
0.60718679593	0.66394594256	0.99422874702
0.60536129816	0.15897329800	0.01025500749
0.60679330862	0.66388051391	0.66088296768

0.60540725640	0.15891955711	0.67678472174
0.60684647580	0.66388190410	0.32789213822
0.60555797773	0.15901368191	0.34404645389
0.60975191810	0.84266308029	0.67729784068
0.61562655925	0.33751489953	0.68240799930
0.60974372805	0.84263924057	0.34391159511
0.61561687319	0.33770282188	0.34857702237
0.60976102198	0.84266362936	0.01101851140
0.61559474698	0.33765140698	0.01572283716
0.87126663910	0.64605411971	0.79923851816
0.87118745012	0.64600567505	0.46604723661
0.87378597974	0.54834731318	0.79903337542
0.87105860008	0.64595333766	0.13266301540
0.87360583091	0.54830306944	0.46550752457
0.87372734750	0.54824883368	0.13218493144
0.00859493102	0.79380980631	0.79929668184
0.00834074796	0.89125688963	0.80480073166
0.00689395502	0.29812351608	0.79908731771
0.00512543356	0.39562926329	0.80472548170
0.00840006285	0.79376623198	0.46577680078
0.00824620750	0.89120727142	0.47133114859
0.00695359016	0.29807579795	0.46516860690
0.00532229034	0.39557361440	0.47161093974
0.00852956055	0.79385931202	0.13199323125
0.00831816815	0.89131094349	0.13752615380
0.00700514557	0.29791647005	0.13236285268
0.00558953260	0.39539991052	0.13848347016
0.37407621295	0.55480705324	0.67264659765
0.36714532155	0.95690403707	0.67381626259
0.36802725323	0.05447110479	0.67394915208
0.37154958454	0.45715894071	0.67213049828
0.37371369024	0.55474842870	0.33987556288
0.36710614932	0.95674395686	0.34067889462
0.36775009875	0.05435430323	0.34030159995
0.37105128121	0.45710631299	0.33905013781

0.37397270201	0.55476461975	0.00592997871
0.36716019724	0.95685363278	0.00759204550
0.36802021457	0.05445915106	0.00770065090
0.37127545942	0.45712552270	0.00559590828
0.49806664622	0.70683490607	0.67299580684
0.49788410394	0.20436310410	0.67574137524
0.49802455257	0.70676648341	0.33969570195
0.49808252070	0.20442371360	0.34311105283
0.49980065647	0.80437399135	0.68048078772
0.50350074005	0.30192309151	0.67873571894
0.49829324870	0.70675929309	0.00616943955
0.49791898203	0.20445080374	0.00930248497
0.49975246414	0.80432782387	0.34705056163
0.50360977195	0.30198600819	0.34539902673
0.49984227343	0.80431317421	0.01350132280
0.50354685955	0.30201943107	0.01214693275
0.94101085867	0.72015850390	0.79769197962
0.94283964728	0.22579666842	0.79606759029
0.94088988414	0.72003403295	0.46435144584
0.94347523013	0.96587073495	0.80165202138
0.94271853743	0.22583515827	0.46266997219
0.93853997387	0.47215177612	0.80196105850
0.94091989063	0.72006442914	0.13085099057
0.94333252688	0.96587775596	0.46826983433
0.94285203601	0.22577774631	0.12921134794
0.93861616358	0.47210538085	0.46815859831
0.94348410063	0.96592374135	0.13496675420
0.93859532975	0.47197981230	0.13502693714
0.43572995271	0.63040625734	0.67299810047
0.43185040678	0.12929688509	0.67491023249
0.43553680078	0.63023612677	0.33969821675
0.43192427908	0.12940491405	0.34178785717
0.43514507149	0.88064266528	0.67870489887
0.43567270020	0.63035090060	0.00622943072

0.43994996906	0.37930759687	0.67634267980
0.43191019872	0.12939628685	0.00866570125
0.43524596085	0.88051576472	0.34538619318
0.43984302761	0.37920990918	0.34281053725
0.43520876706	0.88057443972	0.01185688056
0.43967791927	0.37925546592	0.00952507509
0.88175053205	0.75056041110	0.96194972804
0.87597688738	0.24826785473	0.95968701900
0.88036275504	0.95014933230	0.96717576923
0.87532034097	0.45043287847	0.96786370726
0.88164235550	0.75073978879	0.62888342324
0.87558706357	0.24808137585	0.62715065065
0.88005637727	0.95044875100	0.63378044192
0.87640779718	0.45020274185	0.63398006503
0.88122211360	0.75046313008	0.29508565977
0.87590918908	0.24824010254	0.29315210202
0.87990958183	0.95119235246	0.29985245025
0.87586675043	0.45011330923	0.30020173743
0.03359139580	0.68214193075	0.96794393135
0.02065812469	0.18079176157	0.96638532495
0.03348629624	0.68228736340	0.63500106273
0.03205050968	0.50642354338	0.97007257061
0.02078959388	0.18101116221	0.63304114675
0.02420407916	0.00829567023	0.96958476514
0.03340031633	0.68207684501	0.30146672405
0.03199378807	0.50676771795	0.63662555789
0.02035336083	0.18067404770	0.29968645068
0.02399501086	0.00837003497	0.63600066978
0.03202290966	0.50655528921	0.30317377192
0.02407413086	0.00833299749	0.30306386444
0.37008255871	0.65216041510	0.83739531424
0.36544222794	0.15197429732	0.83917124532
0.37009606022	0.65206733762	0.50426969714
0.36601172305	0.15241196646	0.50567156710

0.36963132042	0.65181647254	0.17057071673
0.37225524912	0.85251619097	0.84321611539
0.36618399029	0.15268968590	0.17279130773
0.37639017264	0.35258010650	0.84184079003
0.37261979841	0.85223450593	0.51127004476
0.37701612487	0.35235235479	0.50868014158
0.37280819278	0.85216379516	0.17748603672
0.37643970833	0.35230089866	0.17524383625
0.53059656243	0.59297562004	0.84251070304
0.52830488153	0.91371584001	0.84652611107
0.52415562047	0.09137274163	0.84538233421
0.53237071335	0.41315787653	0.84479483017
0.53044005858	0.59285871769	0.50904343682
0.52806839476	0.91381009120	0.51283829673
0.52413336661	0.09122095890	0.51170532760
0.53231231160	0.41323809694	0.51109818220
0.53031340213	0.59293031180	0.17574318280
0.52809373401	0.91377566125	0.17972542284
0.52416551992	0.09127754865	0.17880707829
0.53211987055	0.41315070054	0.17790311943

Crystal Structure for bulk of NiHC-4,4-bp-Cl in VASP POSCAR format

1.0000000000

12.31687553000	0.00000000000	0.00000000000
-0.81209881223	22.79390188106	0.00000000000
0.22364194090	-0.15835809866	10.35467045952

C	H	N	Ni	Cl	O	Cl	O
90	84	18	12	6	6	6	6

D

0.15486967982	0.99946869858	0.01731887255
0.15493786988	0.49831603845	0.02136697965
0.65881385879	0.75227843773	0.01585950974
0.15500683949	0.99899564407	0.35088880184
0.15415807716	0.49800275042	0.35519409472

0.65842865395	0.75248151107	0.35106588203
0.15427012893	0.99958648908	0.68365497943
0.15456417301	0.49823796391	0.68923682979
0.65853616574	0.75214561535	0.68464937650
0.15937998204	0.24637226434	0.30811528995
0.15998288869	0.74748826894	0.30305561739
0.65450535363	0.50232676368	0.29942813691
0.15949356297	0.24670953066	0.64160350356
0.16043822602	0.74814032765	0.63679226179
0.65435468945	0.50174152458	0.63264293862
0.16048664934	0.24685150990	0.97544381431
0.16105815194	0.74823246742	0.97008589991
0.65468111098	0.50191218322	0.96609480399
0.15380457024	0.30725377413	0.30615478121
0.15586702102	0.80835646609	0.30033705468
0.65316304208	0.56319620583	0.29545367687
0.15397246609	0.30755302293	0.63972000595
0.15621573119	0.80898879078	0.63380240594
0.65273851758	0.56262981695	0.62899285704
0.15512606762	0.30771534809	0.97376389876
0.15685400236	0.80908826217	0.96823567792
0.65308970471	0.56275181180	0.96150943866
0.15189888522	0.43740058402	0.02278207432
0.15201679262	0.93843965875	0.01849361559
0.65433680607	0.69136761476	0.01358146264
0.15087805822	0.43703021454	0.35609868816
0.15182651192	0.93794008537	0.35169458132
0.65428580001	0.69156036201	0.34934045677
0.15132124942	0.43730177280	0.69001166082
0.15130852171	0.93853146582	0.68441959720
0.65399084183	0.69122592136	0.68301073353
0.24784472028	0.34207988691	0.32918428021
0.24892936340	0.84315867669	0.32879908321
0.74840795369	0.59655368958	0.32209639728

0.24815080706	0.34231797014	0.66273271987
0.24908542359	0.84396565027	0.66228505666
0.74786693668	0.59609230643	0.65554839708
0.24922236412	0.34247870555	0.99670510765
0.24990104040	0.84385695052	0.99680788793
0.74838331163	0.59628770860	0.98742251972
0.24652007739	0.40687211100	0.32958377114
0.24826203228	0.90804206209	0.32935415157
0.74970978880	0.66142242762	0.32207668318
0.24693453783	0.40713019314	0.66335096263
0.24796200044	0.90878129951	0.66240272617
0.74920590915	0.66092431710	0.65540725458
0.24782938285	0.40728748173	0.99709538825
0.24870737391	0.90866918143	0.99709108767
0.74984406758	0.66114624363	0.98728736670
0.33941348499	0.00340585347	0.30977050864
0.33647935370	0.50240458911	0.30629486080
0.84142043262	0.75627859424	0.29952346095
0.33871804941	0.00422237694	0.64295943517
0.33685715124	0.50267288362	0.64018813268
0.84140526975	0.75559707453	0.63245743789
0.33956219389	0.00403278103	0.97726167101
0.33755177430	0.50280234317	0.97401351829
0.84233706234	0.75594433819	0.96715379559
0.34306308470	0.25202312473	0.02034580384
0.34061402378	0.75281131053	0.02637256435
0.83550464903	0.50467534984	0.02023583907
0.34218385441	0.25177256590	0.35248608667
0.33953716657	0.75212962630	0.35898248501
0.83529581281	0.50481959221	0.35412564314
0.34218581661	0.25198126563	0.68657387599
0.33987771355	0.75300484383	0.69307954696
0.83529605947	0.50449732419	0.68654912349
0.34486736094	0.31303201951	0.02011721124

0.34369372689	0.81371330941	0.02499006453
0.84125319024	0.56554569925	0.01648991754
0.34366924142	0.31279443196	0.35237261951
0.34255796139	0.81304816924	0.35748831780
0.84132996454	0.56569262183	0.35073682520
0.34394295543	0.31294838053	0.68580790347
0.34271087751	0.81391815448	0.69140945953
0.84095792491	0.56534554231	0.68390545944
0.34074958435	0.44161146266	0.30406087797
0.34356208586	0.94254210838	0.30869160805
0.84497296806	0.69538089591	0.29677076021
0.34112213759	0.44188548264	0.63783421769
0.34298965037	0.94340458107	0.64133633194
0.84448967369	0.69474172608	0.62951762419
0.34202089674	0.44205191756	0.97191389271
0.34388114323	0.94320318712	0.97616321789
0.84551659548	0.69503357034	0.96341545325
0.08405416321	0.10701091184	0.16937640670
0.10655453804	0.59097902874	0.17398253165
0.08291212133	0.10751401765	0.50387160957
0.10695653277	0.59126781122	0.50755967102
0.08296644937	0.10734877007	0.83769740958
0.10657029831	0.59162237935	0.84091016339
0.08879348668	0.21762338589	0.28894388331
0.09022707485	0.71898860375	0.27891033561
0.58306835076	0.47473124136	0.27721229857
0.08877591876	0.21810154670	0.62202285482
0.09080870646	0.71952846914	0.61267396503
0.58302603879	0.47406798318	0.61051669889
0.08971260265	0.21823859301	0.95630483719
0.09140093451	0.71977972598	0.94531121056
0.58325684395	0.47419830217	0.94448253802
0.07538364165	0.41384179596	0.04551446691
0.07457889766	0.91529922516	0.03829808004

0.57711844936	0.66817294401	0.03489642982
0.07447087054	0.41345942303	0.37921851742
0.07441290946	0.91485798834	0.37171958307
0.57741885653	0.66829828584	0.37213310361
0.07484146347	0.41368831226	0.71284275341
0.07393316639	0.91533061407	0.70394961650
0.57698913946	0.66813853887	0.70584960174
0.07646929212	0.32647981964	0.28490274495
0.08080232949	0.82873317836	0.27405019707
0.57848933733	0.58411008419	0.26938831756
0.07671106898	0.32680962787	0.61813046615
0.08112109785	0.82914549985	0.60694881740
0.57784184546	0.58348619612	0.60343349162
0.07773575922	0.32696410089	0.95280683770
0.08179099744	0.82948782409	0.94223819977
0.57820181508	0.58345392114	0.93560732810
0.08049485628	0.02340985042	0.03298948821
0.08077749993	0.52225355071	0.03975536485
0.58662666197	0.77717712245	0.03733546553
0.08079762485	0.02296091442	0.36783371928
0.08015679929	0.52194612571	0.37455464586
0.58631945997	0.77727926131	0.37349869724
0.07986634750	0.02347492699	0.70007132176
0.08050731764	0.52215351997	0.70849479775
0.58665954071	0.77712366131	0.70711499054
0.41786872005	0.42162767042	0.27998209285
0.42197757147	0.92301512725	0.28967719096
0.92182602287	0.67509856608	0.27276129627
0.41818060905	0.42187918863	0.61348316316
0.42144038742	0.92396577589	0.62197060333
0.92121203968	0.67428096955	0.60537314427
0.41930506200	0.42203133881	0.94882501605
0.42231961270	0.92364567725	0.95768279877
0.92246407460	0.67469711900	0.94003375395

0.41505951970	0.22737002464	0.04003411549
0.41107491163	0.72807091270	0.05132173024
0.90493903448	0.47907418713	0.04511637360
0.41437045817	0.22720251375	0.37150758308
0.41009650707	0.72742945703	0.38371867029
0.90458750314	0.47905178767	0.37865884736
0.41419077433	0.22728878521	0.70607539347
0.41054680297	0.72846601122	0.71804900012
0.90500933471	0.47890726796	0.71028460826
0.41161555043	0.03155603037	0.29291373384
0.40790783431	0.53045219391	0.28559218573
0.91307333278	0.78422737529	0.27887046626
0.41084159091	0.03244226224	0.62628002710
0.40833468158	0.53072520168	0.61975065900
0.91324194060	0.78341195145	0.61168472939
0.41179874426	0.03218050878	0.96041208379
0.40907559268	0.53087839850	0.95424665687
0.91441608276	0.78379009236	0.94787866793
0.42057704338	0.33729715100	0.04090422662
0.41917289082	0.83752111982	0.04861967689
0.91767215728	0.58876921611	0.03903460352
0.41941044602	0.33711826035	0.37261050326
0.41795930820	0.83677205864	0.38146591757
0.91785594161	0.58881492332	0.37312154965
0.41981108586	0.33728541904	0.70536743217
0.41806307626	0.83784340942	0.71511574733
0.91731519655	0.58866173349	0.70623031709
0.59734943694	0.33943089063	0.16944670696
0.57278736289	0.88222843655	0.16938978617
0.59634434983	0.34001038780	0.50207009825
0.57223222442	0.88191963562	0.50343354058
0.59689961307	0.33976853298	0.83492812299
0.57275617630	0.88213539065	0.83743631329
0.24641131912	0.03160642831	0.33008201771

0.24465371878	0.53113239376	0.33120498160
0.74976707471	0.78469007896	0.32670430751
0.24557318753	0.03228482174	0.66344497605
0.24500388286	0.53141388068	0.66506387894
0.75003343859	0.78421635924	0.66007949077
0.24637917466	0.03212161260	0.99683351936
0.24548186056	0.53150537685	0.99798368721
0.75059118841	0.78443170016	0.99334766753
0.25146400476	0.21898957725	0.33123572579
0.24987266984	0.71926148193	0.33276599226
0.74354094926	0.47328959559	0.32920515977
0.25140070660	0.21923423258	0.66524614269
0.25037917602	0.71996834268	0.66669695738
0.74368179541	0.47282876128	0.66188486778
0.25238587916	0.21929639213	0.99856232474
0.25090249825	0.71994629073	0.99993560041
0.74388508435	0.47298373667	0.99560967993
0.24888425043	0.12604694127	0.99831954338
0.24844047229	0.62670896372	0.00146080941
0.24866692550	0.12580037065	0.33166137111
0.24794774435	0.62621279104	0.33434760242
0.24821759970	0.12605162720	0.66526278223
0.24820041898	0.62673906499	0.66771783294
0.74145282662	0.38296453936	0.33137659884
0.74235928567	0.87482580753	0.33093370913
0.74228768191	0.38258567613	0.66451154471
0.74238570282	0.87443831834	0.66456675863
0.74159979330	0.38273553863	0.99817936789
0.74282976736	0.87464333477	0.99783322776
0.87873130655	0.37191139865	0.15978293210
0.87884169575	0.89332335621	0.16237192005
0.87971114251	0.37174890062	0.49290280516
0.87824797103	0.89343485327	0.49575306419
0.87999504871	0.37145712105	0.82659369735

0.87827249258	0.89318358917	0.82875738166
0.15299242174	0.12888742402	0.16784129218
0.14936382183	0.62752926021	0.17220598028
0.15223584066	0.12913733457	0.50121247376
0.14970502461	0.62781213038	0.50425091792
0.15182120272	0.12940271745	0.83515264921
0.14973071020	0.62806992386	0.83843941755
0.39419136435	0.12691628876	0.16119258991
0.39731331261	0.62706794047	0.16233074916
0.39359185644	0.12727007818	0.49412993171
0.39721948018	0.62691422335	0.49553809665
0.39415021673	0.12706358693	0.82746495019
0.39747522191	0.62718900566	0.82876255817
0.64548481926	0.37415726786	0.16826074464
0.65146539387	0.88079105619	0.16628464669
0.64645654244	0.37393757794	0.50039853255
0.65082537317	0.87988396621	0.50039423410
0.64642213063	0.37397666051	0.83451613451
0.65131684932	0.88007605631	0.83457980027
